# Chiral “doped” MOFs: an electrochemical and theoretical integrated study

**DOI:** 10.3389/fchem.2023.1215619

**Published:** 2023-08-08

**Authors:** Rufaro Kawondera, Marco Bonechi, Irene Maccioni, Walter Giurlani, Tommaso Salzillo, Elisabetta Venuti, Debabrata Mishra, Claudio Fontanesi, Massimo Innocenti, Gift Mehlana, Wilbert Mtangi

**Affiliations:** ^1^ Institute of Materials Science, Processing and Engineering Technology, Chinhoyi University of Technology, Chinhoyi, Zimbabwe; ^2^ Department of Chemistry, University of Firenze, Firenze, Italy; ^3^ National Interuniversity Consortium of Materials Science and Technology (INSTM), Firenze, Italy; ^4^ Department of Industrial Chemistry “Toso Montanari”, University of Bologna, Bologna, Italy; ^5^ Department of Physics and Astrophysics, University of Delhi, New Delhi, India; ^6^ Department of Engineering “Enzo Ferrari” (DIEF), University of Modena, Modena, Italy; ^7^ Center for Colloid and Surface Science (CSGI), Florence, Italy; ^8^ Department of Chemical Sciences, Midlands State University, Gweru, Zimbabwe

**Keywords:** metal-organic framework, chiral doping, cyclic voltammetry, solid state electrochemistry, SEM, XRD

## Abstract

This work reports on the electrochemical behaviour of Fe and Zn based metal-organic framework (MOF) compounds, which are “doped” with chiral molecules, namely: cysteine and camphor sulfonic acid. Their electrochemical behaviour was thoroughly investigated via “solid-state” electrochemical measurements, exploiting an “*ad hoc*” tailored experimental set-up: a paste obtained by carefully mixing the MOF with graphite powder is deposited on a glassy carbon (GC) surface. The latter serves as the working electrode (WE) in cyclic voltammetry (CV) measurements. Infrared (IR), X-ray diffraction (XRD) and absorbance (UV-Vis) techniques are exploited for a further characterization of the MOFs’ structural and electronic properties. The experimental results are then compared with DFT based quantum mechanical calculations. The electronic and structural properties of the MOFs synthesized in this study depend mainly on the type of metal center, and to a minor extent on the chemical nature of the dopant.

## Highlights

1. Synthesis of Fe and Zn MOFs doped with chiral molecules
2. Electronic properties via “solid-state” electrochemical measurements
3. Development of a specially adapted electrochemical cell
4. Morphological and compositional characterisation (IR, XRD, SEM, EDS)

## 1 Introduction

Metal-organic frameworks (MOFs) have attracted increasing scientific interest due to unique features including; high specific surface area, high crystallinity, exceptional and tuneable pore size (Ghanbari et al., 2020; Wang and Astruc, 2020). In fact, the opportunity to synthesize porous materials with high modularity and diverse functionality makes MOFs suitable candidates for solid-state materials in electronic applications (Zhao et al., 2020). Moreover, the physical and chemical properties of MOFs can be customized and designed through a suitably tailored synthesis (Li et al., 2016). Thus, due to these special characteristics and exceptional tunability, MOFs differ from traditional porous materials (silica, activated carbon and zeolites) and are promising candidates for application also in sensors and catalysis. In general, MOFs are assembled from metal cations and organic linkers via metal-ligand coordination bonds (James, 2003; Zhou and Kitagawa, 2014). In recent years, scientists have conducted intense research in the production of chiral MOFs (and covalent MOFs, CMOFs) (Gong et al., 2022). In this arena, chiral MOFs are of particular interest because of their specific field of application which includes; chiral enantioselective recognition (Zhao et al., 2017; Han et al., 2019; Niu et al., 2022), enantioselective separation (Padmanaban et al., 2011; Peng et al., 2014; Abbas et al., 2018), asymmetric catalysis (Wang et al., 2011; Bhattacharjee et al., 2018; Dybtsev and Bryliakov, 2021), and sensing (Wanderley et al., 2012; Yang et al., 2021). These peculiar features can be exploited in crystal engineering, optoelectronics, medicine, biology, pharmacology, and environmental science. In particular, chirality within MOFs can be induced exploiting all of the different constituent components, i.e., linker, metal node, or even guest molecules (Ma et al., 2022). The presence of guest molecules embedded within the MOF structure as well as the functionalization of cavities, can induce chiral properties within the overall MOF architecture (Sharifzadeh et al., 2021). In general, there are three ways to obtain chiral MOFs. i) Chirality can be generated during crystal growth thanks to the nature and physical structure of precursors (Altaf et al., 2022). This approach leads to the crystallization of MOFs in a chiral space group because the spatial disposition of achiral building block has an enantiospecific supramolecular interaction. This method shows some difficulties, because the use of achiral components often leads to a final racemization of the system (Zhang et al., 2008). ii) The second method for obtaining chiral MOFs involves the use of chiral likers (Kutzscher et al., 2016). This procedure is a direct method of “chiralization” and there are interesting examples where this strategy results in chiral MOFs showing unique properties (Gedrich et al., 2011; Sawano et al., 2015). On the other hand, the use of chiral linkers may require the development of specific synthesis leading to increased costs and possibly environmental issues due to the substances used. iii) The third approach is an indirect method that leads to the formation of achiral frameworks and utilization of some external chiral agents to produce stereogenic centres (Xue et al., 2016; Zavakhina et al., 2019). Chiral inducing compounds may involve chiral guest, solvent, auxiliary pendant or functionalization (Gheorghe et al., 2021). In this last case the presence of an enantiopure chiral agent is the driving force that induces chirality. This third strategy allows simple, inexpensive molecules to be included in the framework without changing the initial synthesis of the MOF. In electrochemistry, MOFs are a class of promising candidates for electrode surface functionalization and have the potential to extend the application range of electrochemical sensors (Ma et al., 2020). Indeed, many papers can be found in the literature dedicated to the development of MOF-based electrochemical sensors (Guo et al., 2016; Gonçalves et al., 2021; Daniel et al., 2022), in particular for the detection of heavy metal ions in aqueous solutions (Tran et al., 2020). In addition to the previously mentioned fields of application, MOFs are also emerging materials in the field of electrocatalysis (Nivetha et al., 2019; Mukhopadhyay et al., 2020; Yang et al., 2020), thanks to their electrochemical properties (Zhang et al., 2014; Jiang et al., 2021; Yang et al., 2022), which are comparable to those of systems used in water splitting and Oxygen Reduction Reaction (ORR) (Passaponti et al., 2020; Savastano et al., 2020; Bonechi et al., 2021a; Giurlani et al., 2022a). Remarkably, the effective use of chiral surfaces in the water splitting process, concerning the Oxygen Evolution Reaction (OER), has already been demonstrated in the recent past (Mtangi et al., 2015; Mtangi et al., 2017; Garcés-Pineda et al., 2019; Gazzotti et al., 2020). Thus, chiral MOFs appear to be promising materials for use in the water splitting process. Indeed, Fe-based and Zn-based MOFs are a well-known class of compounds. Among the iron-based MOFs, the most common is MIL53(Fe), obtained by a combination between iron(III) cations and 1,4-dicarboxylic acid, eventually yielding a 3D network (or secondary building units) which contain FeO_6_ hexagonal chains and dicarboxylate anions (Sudik et al., 2005; Scherb et al., 2008; Ajpi et al., 2023). MIL53(Fe) shows significant advantages compared with other MOFs, which include chemical stability (Chen et al., 2017; Le et al., 2019), the presence of iron (a nontoxic and widely available metal) and a green and sustainable manufacturing process (Millange and Walton, 2018; Navarathna et al., 2020). Zn-based MOFs are also an interesting class of MOFs as they exhibit structural features similar to those of MIL53(Fe). They have a network consisting of Zn(II) cations tetrahedrally coordinated by two molecules of 1,4-dicarboxylic acid and two molecules of N,N'-bis (pyridin-4-methyl) cyclohexane-1,4-diamine (Altaf et al., 2018).

In this study, Fe based and Zn based MOFs were synthesized and characterised by using spectroscopic techniques: infrared spectroscopy (FT-IR), X-ray diffraction (XRD), and scanning electron microscope (SEM). MIL53(Fe) and Zn MOF derivatives “doped” with chiral molecules were prepared using a simple, ecologically-friendly method, and characterized via X-ray diffraction and spectroscopic experiments. In this work, eight different MOFs are studied. MIL53 (Fe based MOF) where the framework features 1,4-dicarboxylic acid as linker, is named (MIL53). MIL53 (Fe based MOF) where the framework features aminoterephthalic acid as linker, is named NH2 MIL53. MIL53 (Fe based MOF) with a moderate addition of L-cysteine, R-camphorsulfonic acid and S-camphorsulfonic acid, are named MIL53 L-Cys, MIL53 R-CSA and MIL53 S-CSA, respectively. A Zn based MOF where the framework features 1,4-dicarboxylic acid and N′-N bis(pyridin-4-ylmethylene)cyclohexane-1,4-diamine (bpcda) as linkers, is named (Zn-MOF). A Zn based MOF where the framework features aminoterephthalic acid as linker, is named (NH_2_-ZnMOF). Zn based MOFs with a moderate addition of L-cysteine, R-camphorsulfonic acid and S-camphorsulfonic acid, are named Zn-MOF L-Cys, Zn-MOF R-CSA and Zn-MOF S-CSA, respectively. L-cysteine, R-camphorsulfonic acid and S-camphorsulfonic acid were selected as the preferred chiral linkers because of their small chain length.

In particular, the electrochemical behaviour was studied by performing cyclic voltammetry (CV) measurements using a solid-state electrochemical approach (Solano et al., 2021; Solano et al., 2022). Typically, electrochemical-based CV measurements are exploited to study the electrochemical behaviour of metals and organic compounds in bulk solution (Bonechi et al., 2021b; Stefani et al., 2021; Giurlani et al., 2022b; Bonechi et al., 2022), or using commercial systems of the electroplating industry (Berretti et al., 2020; Mariani et al., 2022; Comparini et al., 2023). In this study we adopted a new custom-designed setup to probe the crystal electrochemical behaviour, to avoid the dissolution of the solid-state material. The experimental results have been compared with data obtained by using DFT based quantum mechanical calculations.

## 2 Experimental

### 2.1 Chemicals

All chemicals and reagents were analytical grade. N′N dimethylformamide (DMF) and Zinc Nitrate were purchased from ACE Chemicals, South Africa. Titanium (IV) oxide nanoparticles (21 nm primary particle size), Fluorine doped tin oxide (FTO) (surface resistivity of ∼7Ω/sq) coated glass substrates, ethanol, acetone, Iron(III) chloride FeCl_3_·6H_2_O, terephthalic acid H_2_BDC (benzene-1,4-dicarboxylic acid), 2-aminoterephthalic acid H_2_N-BDC (2-aminobenzene-1,4-dicarboxylic acid), ethanol, L-cysteine, R-camphorsulfonic acid, S- camphorsulfonic acid, acetonitrile, graphite powder (<20 µm) and potassium chloride (KCl) were purchased from Sigma, Germany and used with no further purification. Aqueous solutions were prepared using milliQ water.

### 2.2 Instrumentation

Original Fe and Zn based doped MOFs (eight different compounds) were synthesized in a Digital oven, Labotec EcoTherm Economy Oven, South Africa. Details concerning the synthesis are reported in the Supporting Information. Infrared measurements were performed using the FT-IR spectrometer, Perkin-Elmer Spectrum Two, United States, equipped with a transmission mode interferometer Dynascan, Perkin-Elmer, United States. IR spectra were recorded in the range 400–4,000 cm^−1^ with a spectral resolution of 2 cm^−1^ and 32 scans. Powder X-ray diffraction (PXRD) measurements were carried out using a Bruker D2 phaser XRD machine, United States, with DIFFRAC-SUITE software for controlling the instrument and analyzing the data. Scanning electron microscope (SEM) images were investigated using a Scanning Electron Microscope, SU3800, Hitachi, Japan, equipped with an EDS detector UltimMax, Oxford instrument, United Kingdom. For PXRD measurements MOF were immobilised on a copper surface by dropcast of an acetonitrile dispersion dried in a nitrogen stream. UV-Vis spectra were recorded by using a UV-1900i Shimadzu UV-Vis Spectrophotometer using 1 cm path length polystyrene cuvette for photocatalytic measurements. CH Instruments Electroanalyser model 608E and electrochemical workstation (potentiostat/galvanostat) PGSTAT204, Metrohm AutoLab, Switzerland, were used to perform electrochemical measurements, driven by Nova2.1 software.

### 2.3 Synthesis of MOFs

#### 2.3.1 Synthesis of MIL53 and NH2 MIL53.

1.35 g of FeCl_3_•6H_2_O and linkers were dissolved in 25 mL DMF. The amount of linker corresponding to 0.85 g of H_2_BDC for the synthesis of MIL53 and 0.93 g of NH2-BDC for the synthesis of NH2 MIL53. The mixture was then transferred into a Teflon-lined autoclave and heated up at 150°C for 6 h. The yellow solid was extracted, refluxed with DMF for 12 h. After filtering the solid was washed with C_2_H_5_OH for three times (3 mL × 10 mL), and then dried at 80°C.

#### 2.3.2 Synthesis of chiral MIL53 derivatives

In the case of chiral derivatives, 0.04 g of L-Cysteine was added to the reaction vessel for the synthesis of MIL53 L-Cys, 0.06 g of R-camphorsulfonic acid for the synthesis of MIL53 R-CSA, 0.06 g of S-camphorsulfonic for the synthesis of MIL53 S-CSA. The procedure used for the synthesis of MIL-53 was used for the synthesis of the chiral MIL-53. The quantities for FeCl_3_•6H_2_O, H2BDC and DMF remained the same in all the MIL-53 derivatives.

#### 2.3.3 Synthesis of Zn-MOF

To obtain the Zn-MOF, the synthesis of the linker agent N,N’-bis(pyridin-4-ylmethyl)cyclohexane-1,4-diamine (BPCDA) was first carried out. 4-Pyridylcarboxyldehyde (5 g) and (1R,4R)-cyclohexane-1,4-diamine (5.33 g) was added to ethanol (30 mL). Then, triethylamine (7.08 g) was added slowly and refluxed for 4 h. After refluxing, the reaction mixture was cooled down to room temperature. Portion-wise addition of NaBH_4_ (4.41 g) was carried out and then the stirring of reaction content was performed overnight at room temperature. 10 mL of water was added to quench the extra reducing agent, while 40 mL of dichloromethane (DCM) was added to separate the organic layer. Repeated extractions with DCM were carried out and the organic layers were combined. The product was dried by removing the organic solvent by using anhydrous Na_2_SO_4_ through evaporation. Then the synthesis of the zinc-based metal organic framework was carried out. A mixture of Zn(NO_3_)_2_·6H_2_O (0.297 g, N,N’-bis(pyridin-4-ylmethylene)cyclohexane-1,4-diamine (bpcda) (0.297 g), H_2_BDC (0.172 g) and a mixture of DMF and H_2_O (10 mL, v/v: 3/1) was prepared and placed in glass vial. The vial was loosely capped and heated at 105°C for 3 days. White block-shaped crystals were obtained after which the vial was cooled to room temperature, washed in DMF three times (3 × 10 mL DMF) and dried in air.

#### 2.3.4 Synthesis of chiral Zn-MOF derivatives

In the case of Zn-MOF chiral derivatives, 0.0121 g of L-Cysteine was added to the reaction vessel for the synthesis of Zn-MOF L-Cys, 0.0232 g of R-camphorsulfonic acid for the synthesis of Zn-MOF R-CSA, 0.0232 g of S-camphorsulfonic for the synthesis of Zn-MOF S-CSA. The procedure used for the synthesis of ZnMOF was used for the synthesis of the chiral Zn based MOFs. The quantities for Zn(NO_3_)_2_·6H_2_O, bpcda, H_2_BDC and solvent (DMF and water) remained the same in all the Zn based MOF derivatives.

### 2.4 Electrochemical measurements

The MOF electrochemical behaviour was investigated within the so-called solid-state electrochemical paradigm by using an in-house flat-cell of original design in a typical three-electrodes electrochemical cell set-up. The GCE/MOF served as the working electrode (WE), while a coiled Pt wire and an Ag/AgCl (saturated KCl) electrode served as the counter (CE) electrode and reference electrode (RE), respectively. The special flat-cell configuration of the cell, developed in the past for ECALE depositions (Forni et al., 2000; Innocenti et al., 2001; Giurlani et al., 2018; Salvietti et al., 2018; Giurlani et al., 2020) and adapted for solid-state measurements, features the working electrode placed at the bottom, to help maintaining the graphite-powder/MOF mixture in position. A suitable arrangement was adopted (Supplementary Figure S1), a cylindrical Teflon cell featuring a hole (7 mm diameter) in the bottom was used in a vertical configuration, where the glassy carbon electrode (GC) (1.5 cm diameter) was tightened from below. The GC electrode is held in place by a 1.5 cm diameter screw, which also ensures the electrical contact. A Teflon ring was used to ensure no solution leakage from the cell. The CE and RE were placed at the top of the cylinder in contact with the electrolytic solution. Prior to surface modification, the GC working electrode was polished using 0.05 μm alumina slurries on a polishing cloth; it was cleaned carefully, and then sonicated for 10 min in milliQ water to clean the surface and remove any polishing residue. Finally, the GC working electrode was dried in a nitrogen stream. The effective cell set-up and GC electrode activation procedure was cross-checked by recording CVs in a solution of 1 mM ferricyanide in 0.1 M KCl, in the −0.3 to +0.7 V potential range, we obtained a ΔEp (peak-to-peak separation between the oxidation and reduction potentials) at 25 mVs^−1^ of 60 mV. MOFs crystals (1.0 mg) were placed over the WE and covered with of graphite powder (10.0 mg). A drop of acetonitrile is added to make the mixture compact and then the GC modified electrode was left “to dry” for 10 min in a nitrogen atmosphere. CV measurements were recorded in the −0.8 to +1.2 V potential range vs. Ag/AgCl KCl _sat_. Throughout the paper all the potential values are expressed with reference to the Ag/AgCl/KCl_sat_ reference electrode (RE), unless otherwise stated.

### 2.5 Preparation and functionalization of FTO/TiO_2_/MOFs electrodes.

TiO_2_ nanoparticulate films were deposited on fluorine-doped tin oxide, FTO (surface resistivity of ∼7 Ω/sq) coated glass, using the electrophoretic deposition (EPD) technique. A suspension of TiO_2_ nanoparticles (NPs) was prepared by dispersing 0.4 g TiO_2_ NPs in 40 mL of de-ionized water. Prior to making dispersions, TiO_2_ nanoparticle powders were heated at 570 K for 1 h. The mixture was stirred overnight to ensure homogeneity. Prior to nanoparticle deposition, the FTO substrates were boiled in acetone for 15 min, followed by 15 min of boiling in ethanol, and finally rinsed with de-ionized water. After rinsing, the substrates were dried in the air for 15 min. Electrophoretic deposition (EPD) was performed with a CHI electrochemical analyser, model 608E, using the Chronopotentiometry mode. During EPD, the suspension was continuously stirred using a magnetic stirrer. After completion of the last cycle, the electrodes were annealed for 2 h at 400K. Functionalization of FTO/TiO_2_ surfaces to yield the final FTO/TiO_2_/MOF photoelectrodes was achieved by drop casting 20 µL of 1 mM slurry MOF solutions dissolved in ethanol onto the FTO/TiO_2_ surfaces. The electrodes were then left to dry under controlled humidity for a period of 3 weeks.

### 2.6 Theoretical calculations

Calculations were performed in the framework of *ab initio* quantum mechanical based methods with Gaussian (Frisch et al., 2017) and Quantum Espresso (Giannozzi et al., 2009) programs, using C1 symmetry and unrestricted wave function. Chemcraft (Andrienko, 2023) is used to display molecular structures and molecular orbitals. Molecular orbitals were obtained by full optimization carried out at UB3LYP/6-31G(d) levels of theory. Geometry optimization was carried out by using Barone and Cossi’s polarizable conductor model (CPCM) (Cossi et al., 2003) to account for N,N-dimethylformamide interaction.

## 3 Result and discussion

### 3.1 Solid-state cyclic voltammetry

**FIGURE 1:**
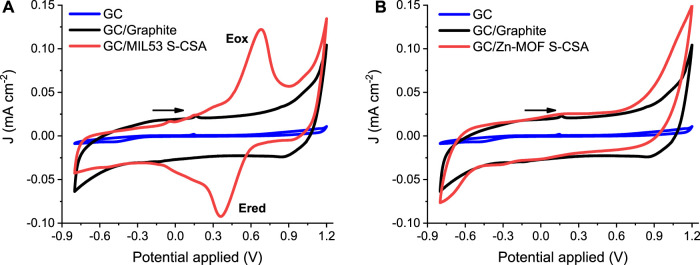
CV curves in aqueous 0.1 M KCl, Pt (CE), Ag/AgCl/KCl_sat_, 10 mV s^−1^ the potential scan rate: **(A)** GC/MIL53 S-CSA (WE) **(B)** GC/Zn-MOF S-CSA (WE).

Figures 1A, B show CV curves recorded for the chiral “doped” MOF MIL53 S-CSA and Zn-MOF S-CSA, respectively. The black solid line is the control CV recorded using graphite powder only, i.e., the “blank” baseline. It must be noted that a prominent capacitive contribution in the—0.7–1.0 V potential range yields a quite regular “rectangular box” CV pattern as depicted in Figures 1A, B. The red solid curves in Figures 1A, B are the CVs recorded after mixing the graphite powder with the chiral MOF (details of the electrode preparation are in the experimental section). The CV of the MIL53 S-CSA MOF features two redox peaks (labelled as Eox and Ered in Figure 1A) centred at about 0.5 V. This suggests that the redox process underlying the presence of the two current peaks in Figure 1A can be assigned to the Fe (III)/Fe(II) redox couple. The peak-to-peak separation is about 0.3 V, which is rather large, such a potential difference is due to the solid-state nature of the electroactive system. Moreover, iron is in an octahedral coordination (a quite stable state environment).

In addition, the overall MOF framework is based on organic molecules which are well known to behave more closely to a dielectric than to a conductive material, this further hinders the Fe(III)/Fe(II) charge transfer kinetics. Supplementary Figure S2 shows the CV curves obtained for the remaining four Fe based MOFs studied in this paper and Supplementary Figure S3 shows the CV curves for ferrocene a well-studied material used as a control and compared with MIL53 S-CSA. Table 1 summarizes the electrochemical results.

**TABLE 1:** Electrochemical results, iron based MOFs.

Compound	Oxidation	Reduction	Difference
	(V)		
MIL53	0.682	0.366	0.316
MIL53 S-CSA	0.676	0.363	0.313
MIL53 R-CSA	0.608	0.418	0.190
NH2 MIL53	0.592	0.408	0.184
MIL53 L-Cys	0.805	0.198	0.607

Please note the effect of “doping” due to the presence of different organic “dopants”. The selected four dopants actually affect the electrochemical behaviour by both shifting the redox peak potential and CV pattern. CSA derivatives induce an overall shift of the peak potential to slightly more positive values, featuring a much prominent quasi-reversible behaviour. Current peaks are definitively much more symmetric (compare Figure 1A main; Supplementary Figure S2C Supporting Information). A rather peculiar result is obtained in the case of cysteine, where a much broader shoulder is present. This is probably due to the overlap of two/three separate peaks, which suggests the existence of two/three different species formed between iron and cysteine (cysteine features three functional groups able to bind iron, i.e. the thio, amino and carboxylic acid moieties). The electrochemistry of Zn based MOFs is characterized by the absence of any reversible (or quasi-reversible) oxidation/reduction current signal, as it can be seen by the inspection of the CVs shown in Figure 1B, Supplementary Figure S4 in the Supporting Information. In this respect Zn based MOFs appear promising materials, to be used in amperometric sensors, because of very low faradaic currents and large volume/surface ratio. On the other hand, the absence of any faradaic current is a direct proof of the electrochemical stability of the “doping” chiral compounds, cysteine and camphor sulphonic acid.

### 3.2 Vibrational IR spectroscopy results

**FIGURE 2:**
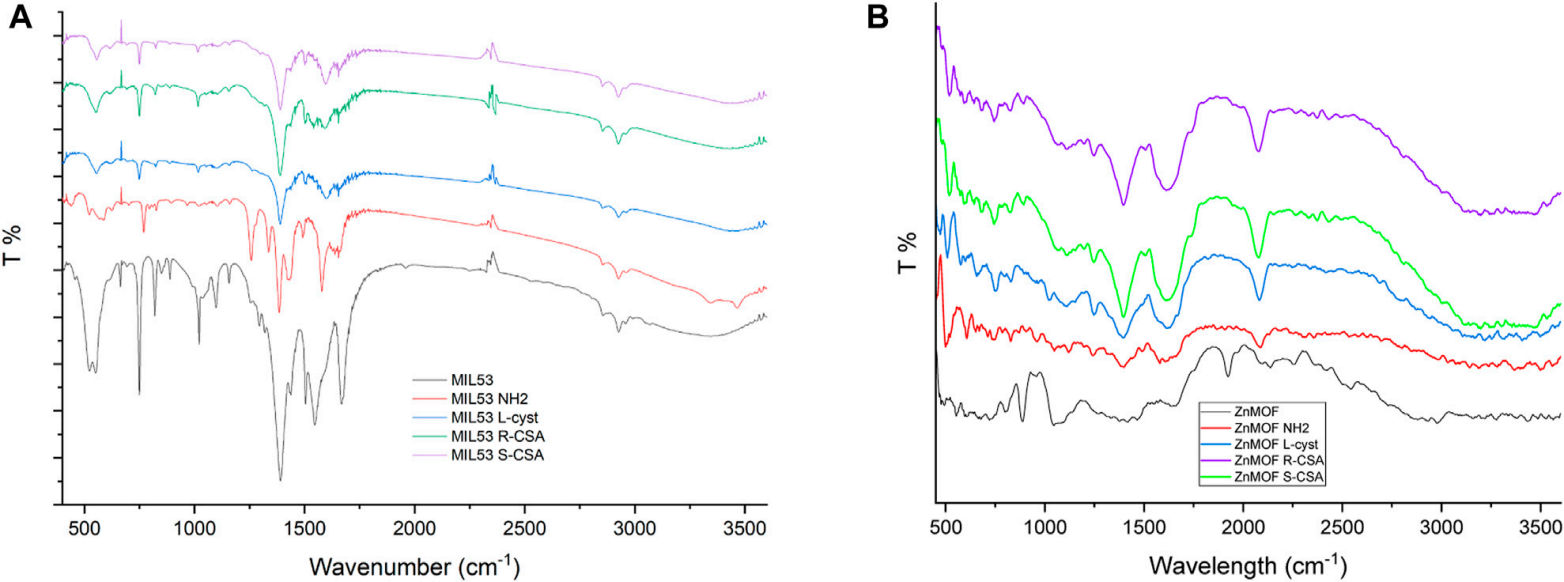
**(A)** Infrared spectra of MIL53 and chiral MIL53 derivatives MOFs recorded in KBr pellet. **(B)** Infrared spectra of Zn-MOF and chiral Zn-MOF derivatives recorded in KBr pellet.

Figure 2A shows the IR spectra of the reference MIL-53(Fe) MOF and chiral MIL-53(Fe) based MOFs, synthesized by including chiral linkers. The IR spectrum of MIL-53 (black line) shows the characteristic absorption peaks of MIL53(Fe) shows the characteristic absorption peaks of MIL53 were obviously observed at 1,668, 1,546, 1,391, 750, and the doublets 552/523 cm^−1^. These values are in agreement with those reported in the literature for iron-based MOFs (Banerjee et al., 2012). In detail, the peak observed at 1,668 cm^−1^ is assigned to the carboxylic C=O bonds stretching vibration confirming the absence of free ligand in the synthesized MIL53 sample. Indeed, the free ligand MIL53 precursor H_2_BDC shows the carboxylic C=O stretching at about 1,680 cm^−1^, the decrease in energy confirms the interaction of the ligand with the Fe of the network (Li et al., 2018). The two intense peaks charactering the IR spectrum at 1,546 and 1,391 cm^−1^ instead correspond to the asymmetric and symmetric vibrations of the C-O of the two carboxyl groups of the H_2_BDC ligand, respectively. The intense and sharp peak at 750 cm^−1^ is assigned to the aromatic C-H bending of the benzene ring. These results confirm the presence of the dicarboxylate linker within frameworks (Yang et al., 2016). The bands in the fingerprint region, between 700 and 400 cm^−1^, are ascribed to the stretching vibration of Fe–O, indicating the formation of a Fe-oxo bond between the Fe(III) and the carboxylic group of BDC ligand (Gong et al., 2002). NH2 MIL53 shows the same spectral pattern and more features in the range 3,300–3,500 cm^−1^ which belongs to the N-H stretching of the amino group of the amino-terephthalic acid used for its synthesis. The presence of the amino group on the benzenic ring of the terephthalic acid shifts the C-H bending peak from 750 cm^−1^ of the MIL53 to higher energy at 769 cm^−1^. MIL53 MOFs synthesized with chiral ligands, L-cyst, R-CSA and S-CSA, all show the same peaks as described above with the confirmation of the Fe-O bond formation by the peak at 552 cm^−1^. The IR spectra of the Zinc based MOFs (Figure 2B) shows the typical structure due to the carboxylic moiety in the 1,300 to 1,600 cm^−1^ range, even if Zn MOFs spectra are not as well defined as the MIL53 ones. A peak in the fingerprint region, at 431cm^−1^ can be attributed to the Zn-O bond, the peaks observed at 811 cm^−1^, and 1,043 cm^−1^, can be N-H wag and a C-N stretch. ZnMOF-NH_2_ and ZnMOF L Cysteine show a peak between 3,300 cm^−1^, and 3,400 cm^−1^, which can be attributed to N-H stretching. Of interest is, when comparing the Zn MOF spectra and the derivatives, the peak observed at 1923 cm^−1^ has undergone a red shift and can now be observed at 2081cm^−1^ in Zn MOF S-CSA and Zn MOF L Cysteine. This shift can be attributed to the formation of hydrogen bonds between the chiral linkers and the linker N,N'-bis(pyridin-4-methyl)cyclohexane-1,4-diamine.

Indeed, there is also a neat difference in the morphological aspect between the iron based and zinc based MOFs; and the crystal structure of the Zn based MOFs is definitively much more disordered than that of the iron based ones.

### 3.3 Structural results

Since its first synthesis MIL53 has shown some analogies with the other MIL-MOFs based on other metallic cations such as Cr^3+^ (Serre et al., 2002) and Al^3+^ (Loiseau et al., 2004) materials MIL53(Cr) and MIL53(Al) but X-ray showed that they are not isostructural. MIL53(Fe) is built up from corner-sharing trans chains of octahedral Fe linked together by benzene-dicarboxylate (BDC) moieties forming an open framework with channels parallel to the crystallographic c-axis. MIL53 shows a peculiar nature presenting a reversible phase transition between the hydrated and the dehydrated form at near room temperature (below 330 K) (Millange et al., 2008).

**FIGURE 3:**
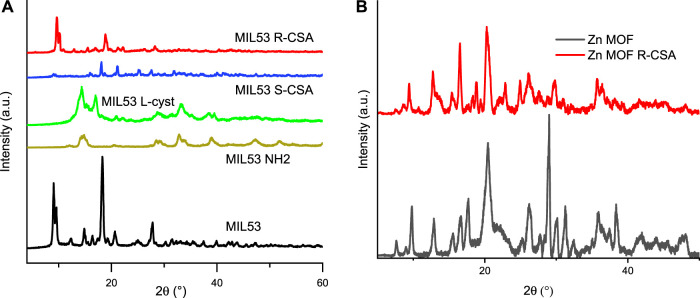
Powder XRD. **(A)** MIL53 and MIL 53 R-CSA and comparison with the simulated ones from literature single crystals structures of the MIL53 in the hydrated and dehydrated form **(B)** Zn-MOF and Zn-MOF R-CSA.

Figure 3 sets out X-ray diffraction pattern of iron and Zn MOFs. In particular, Figure 3A reports the comparison between the powder XRD of the MIL53 and MIL53 R-CSA synthesized in the present work and the simulated ones form the literature crystal structure of the two hydrated and dehydrated form of MIL53. MIL53 and MIL53 R-CSA synthesized in this work appear to be a mixture of dehydrated form, as for the presence of the diffraction peak at about 12.7 (°), and of the hydrated form, for matching the peak at 19 (°). Indeed, the reference MIL53 and the CSA doped MOFs result rather similar. From the tight comparison of XRD patterns shown in Figure 3, we can infer that iron is in its usual octahedral (complexed by oxygen) site coordination, and that CSA does not destroy or alter in a significant way the MOF framework, but rather occupies interstitial free space within the MOF framework (see Supplementary Figure S5 for the molecular iron and zinc based MOFs structure).

### 3.4 SEM and EDS composition

**FIGURE 4:**
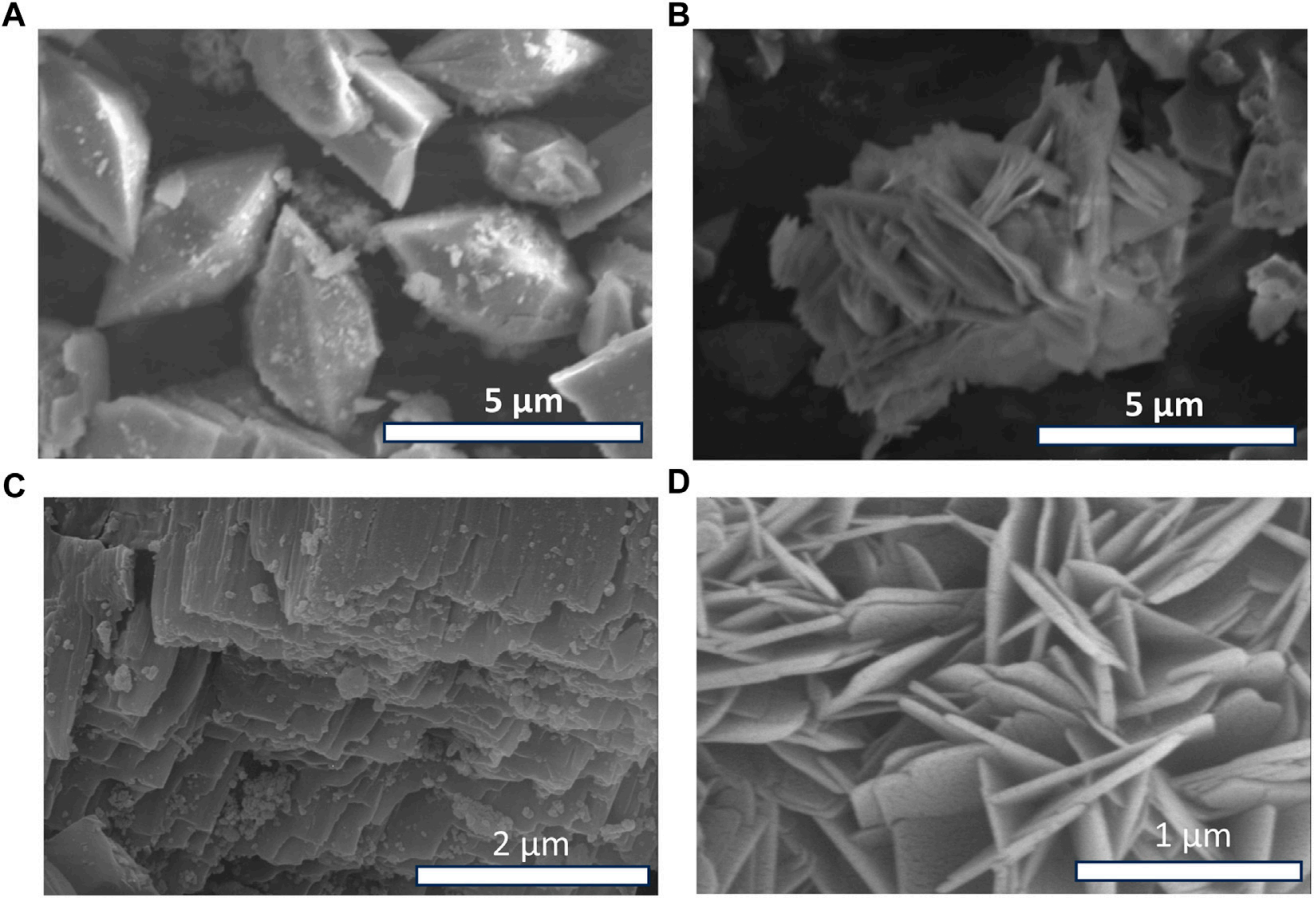
SEM images: **(A)** MIL53 **(B)** Zn-MOF. High resolution FESEM images: **(C)** MIL53 **(D)** Zn-MOF.

Figure 4 shows SEM images of iron-based MIL53 and zinc-based MOF. In the two cases the morphology appears different. MIL53 shows a regular morphology while Zn-MOF shows a lamellar morphology. In both cases, the presence of structures of less than 5 µm is observed. The chiral variants of MIL53 show similar structures to that of MIL53 while NH2 MIL53 is instead organised in more spherical structures, all images are shown in Supplementary Figures S6–S10. Zn-MOF R-CSA and Zn-MOF S-CSA show the typical lamellar structure of Zn-MOF differently, the lamellar structure is less visible in NH2 Zn-MOF and Zn-MOF L-Cys. All SEM images of Zn-MOF are shown in Supplementary Figures S11–S15. EDS spectra performed on the MOF powder are shown in Sections 6 and 7 of the supporting information. In particular, EDS spectra make it possible to find the chemical composition of MOFs (expressed as an atomic percentage). Note that in the MIL53-type structures, the presence of iron is noted. In the chiral variants MIL53 L-Cys, MIL53 R-CSA, MIL53 S-CSA, EDS analysis confirms the presence of sulphur (element present in cysteine and camphor sulfonic acid doping molecules). Similar considerations can be made for Zn-MOF, in which Zn is observed. The EDS analysis on the chiral variants Zn-MOF L-Cys, Zn-MOF R-CSA and Zn-MOF S-CSA show the signal related to the presence of sulphur. Figures 4C, D show high resolution FE-SEM images of MIL53 and Zn-MOF, respectively. In the case of MIL53 emerges a morphology rather different from the low resolution one: the octahedral-like shape of the MIL53 crystals show a lamellar substructure revealing a layer substructure.

### 3.5 Theoretical results

**FIGURE 5:**
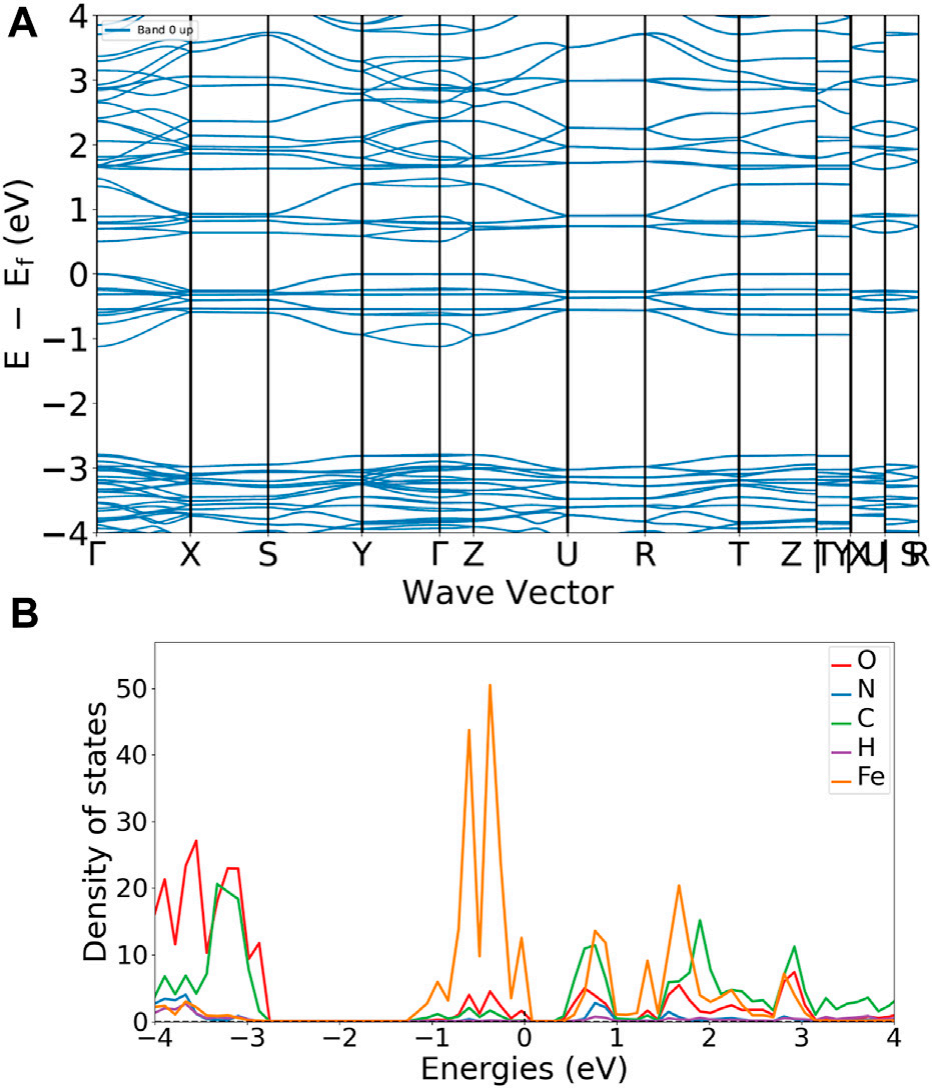
MIL53 reference, quantum espresso results. **(A)** band *versus* wave vector plot. **(B)** projected density of state for the atoms in the MIL53 MOF framework.

Quantum mechanical based calculations are carried out to compare the electronic structure information gained by the electrochemical analysis as a function of the MOFs molecular structure, Figure 5. Calculations have been performed using, both localized orbitals and plane wave DFT methods. Figure 5A shows a typical band energy *versus* wave vector plot. Focusing on the boundary between the valence and conduction band (close to the Fermi energy, Ef) a band gap of about 0.5 eV is found, which is in reasonable agreement with the results obtained in the case of solid state electrochemistry investigations dealing with organic charge transfer crystals (Solano et al., 2021; Solano et al., 2022). The relevant density of state (DOS) plot, Figure 5B, gives due reason to the role of iron in the CV results. In that, the projected DOS (pDOS) plot shows a maximum localization on iron. Please compare the orange peaks around the Fermi energy.

Also localized orbitals calculations yield a picture widely in agreement with results obtained by using plane wave basis with periodic boundary constraints. The Mulliken net charge on iron is positive and both the HOMO and LUMO are delocalized between the centra iron and the oxygens of the carboxylic groups coordinating the iron (compare Section 8 of the supporting information). In tight agreement with the experimental electrochemical results which indicate that the quasi-reversible current peaks evident in the CV curves are due to the iron oxidation in the forward curve.

### 3.6 Photo-electrochemical water splitting

**FIGURE 6:**
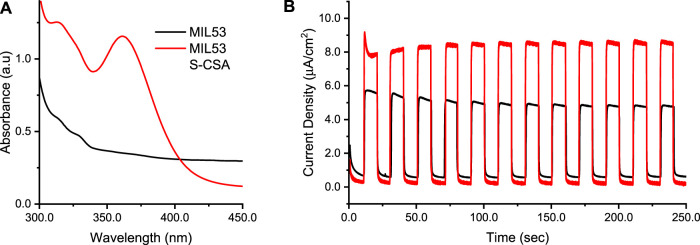
Photoelectrochemical results of FTO/TiO_2_/MIL53 and FTO/TiO_2_/MIL53 S-CSA working electrodes, Pt wire the CE, Ag/AgKCl_sat_the RE, 0.1 M sodium sulphate aqueous solution. **(A)** UV-Vis spectra: black line the MIL53, red line the MIL53 S-CSA. **(B)** Chronoamperometry recorded at constant 1.0 V potential under chopped, 20 s for the whole on/off single cycle, AM1.5g illumination: black line the MIL53, red line the MIL53 S-CSA.

The MOFs synthesized here, supported on titania, were used as working electrode in the water splitting process, in a photo-electrochemical experiment. In particular, MOFs supported on titania served as the working electrode (anode) for the oxygen evolution reaction (OER). MIL53 S-CSA was selected as a candidate for photo-electrochemical measurements, because of CV and UV-Vis results. UV-Vis spectra of MIL53 and Zn-MOF are reported in Supplementary Figures S16, S17 respectively. Chronoamperometry curves (current vs. time) at constant 1.0 V potential under illumination of an AM1.5G solar simulator were recorded. Supplementary Figure S18 shows the Air Mass (AM) experimental spectrum used as excited light source in photoelectrochemical experiments. To investigate the effect of photo-excitation, dark and light conditions were obtained by chopping the light source ON and OFF, in 20 s intervals between light-ON and light-OFF single cycles. Figure 6A sets out the absorption UV-Vis spectra of both MIL53 (black line) as a reference, and the chiralized MIL53 S-CSA (red line), Figure 6B shows current vs. time chronoamperometry curves. 1.0 V was selected as a potential close to the onset of the OER, but still without oxygen evolution. Figure 6B shows that the chiralized MIL53 S-CSA is characterized by a substantial increase in the photocurrent (light on) with respect to the achiral one. On one side, the advantage of the chiralized MIL53 S-CSA is due to the larger absorption with respect to the achiral MIL53, as depicted in Figure 6A. MIL53 S-CSA takes advantage of both being a more efficient light antenna and as a spin-filter (being chiral) as a result the OER reaction is catalyzed. All in all, this outcome is consistent with results present in the literature, where the redox processes concerning the oxygen are shown to be sensitive to the handedness of the electrode surface via chirality-spin interaction (Mtangi et al., 2015; Mtangi et al., 2017; Gazzotti et al., 2020; Sang et al., 2022).

From literature cited above, it has been noted when chiral photoelectrodes are used in photoelectrochemical water splitting, there is spin filtering of the transmitted electrons i.e., there is preferential transmission of electrons spinning one direction. When there is electron spin filtering, the formation of triplet oxygen (^3^O_2_) is promoted, while formation of hydrogen peroxide is minimised. When the formation of hydrogen peroxide is minimized the yield of hydrogen improves, this can also be correlated to the current densities produced by achiral MIL-53 vs. MIL53 doped with R CSA.

**FIGURE 7:**
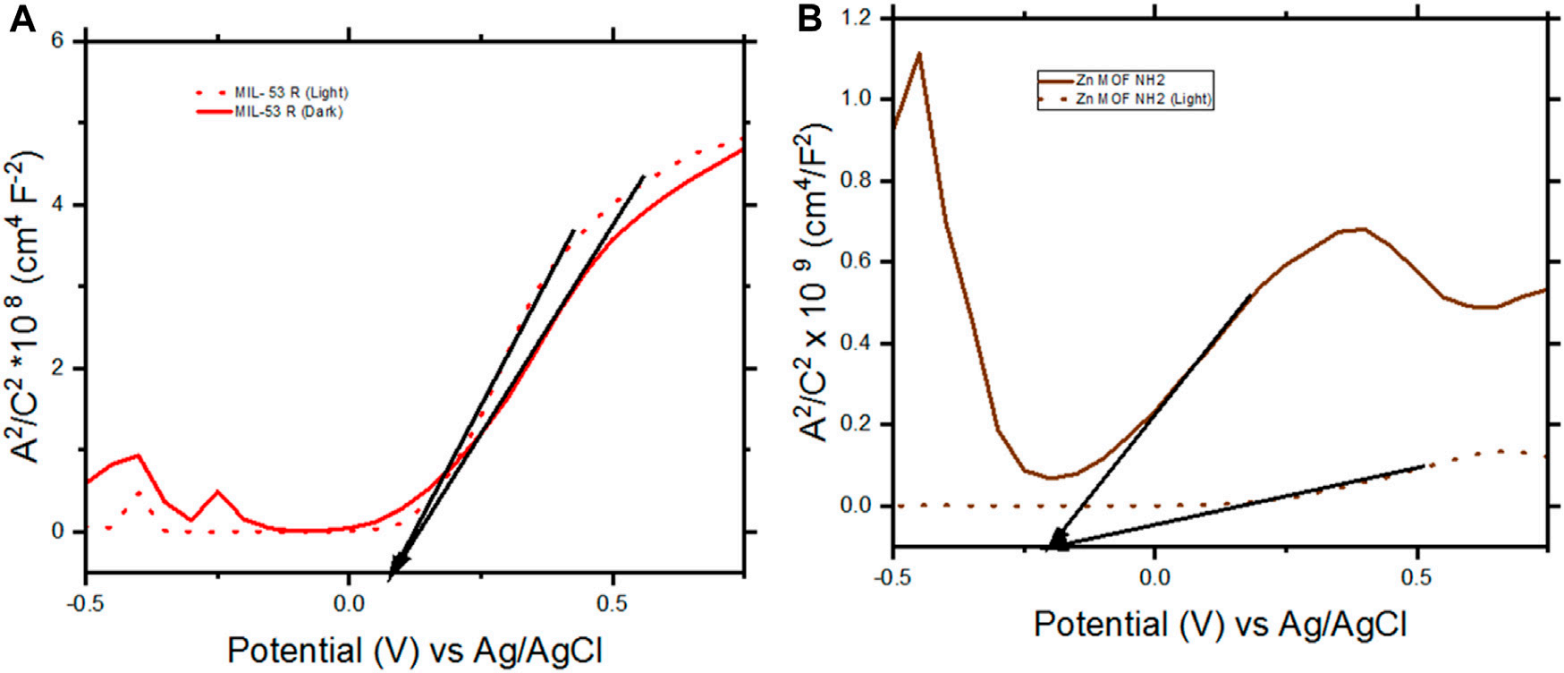
Mott- Schottky results of FTO/TiO_2_/MIL53 R-CSA and FTO/TiO_2_/Zn MOF NH_2_ working electrodes. **(A)** FTO/TiO_2_/MIL53 R-CSA. **(B)** FTO/TiO_2_/Zn MOF NH_2_. The measurements were performed while sweeping voltages from 0.75 V to −0.5 V using an AC Voltage with an amplitude of 5 mV and frequency of 1,000 Hz, under illumination and under darkness.

During water splitting, Mott-Schottky analysis was carried out to determine the electronic properties of the photoelectrodes (Figure 7). The measurements were performed while sweeping voltages from 0.75 V to −0.5 V using an AC Voltage with an amplitude of 5 mV and frequency of 1000 Hz. Measurements were carried out under illumination and under darkness.

The flat band potentials for MIL 53 R-CSA under illumination and under darkness are similar at + 0.08 V, while for Zn MOF NH_2_ the flat band potentials are – 0.19 V, due to the fact that the flat band potentials are similar, we can conclude that the molecules did not affect the electronic properties of the TiO_2_ and that the observed current densities, were due to the activity of the molecules deposited.

## 4 Conclusion

MOFs of the MIL53 class have been synthesized and “doped” with a small amount of four different organic compounds: achiral amino terephthalic acid, enantiopure S-CSA, R-CSA and L-cysteine. Structural characterization indicates that the organic “dopants” do not interfere with the MOF framework, but rather occupy interstitial sites within the MOF framework, i.e. a sort of “supermolecular” architecture. Nonetheless, “dopants” affects the electronic structure of the MOF, as it is suggested by the variation of redox potentials of the Fe(III)/Fe(II) couple in CV curves. Eventually, DFT theoretical calculations yield an overall picture in agreement with these experimental evidences. It is definitively worth of further investigation the “strong” influence of L-cysteine on the electronic properties of the MOF, as it is substantiated by the major variation in the CV redox current peaks, yielding a splitting of the peak into two broad swallow peaks. Eventually, photoelectrochemical results show that the “chiral” doping, MIL53 S-CSA, leads to a substantial increase in the OER photocurrent, this result seems due to the role of chiral electrode surfaces in the oxygen redox process. (Mtangi et al., 2015; Mtangi et al., 2017; Sang et al., 2022).

## Data Availability

The raw data supporting the conclusion of this article will be made available by the authors, without undue reservation.
