# Supplementary material for: Chiral “doped” MOFs: an electrochemical and theoretical integrated study
Source: Front Chem. 2023 Aug 8;11:1215619. doi: 10.3389/fchem.2023.1215619 (PMC10442718; doi:10.3389/fchem.2023.1215619)
Supplement: Supplementary file 1 [file DataSheet1.docx]

**Supporting information**

**Chiral “doped” MOFs: An electrochemical and theoretical integrated study**

Rufaro Kawondera^1,†^, Marco Bonechi^2,†^, Irene Maccioni^2^, Walter Giurlani^2,3^, Tommaso Salzillo^4^, Elisabetta Venuti^4^, Debabrata Mishra^5^, Claudio Fontanesi^3,6^, Massimo Innocenti*^2,3,7^, Gift Mehlana^8^, Wilbert Mtangi*^1^

*^1^Institute of Materials Science, Processing and Engineering Technology, Chinhoyi University of Technology, P Bag 7724, Chirundu Road, Chinhoyi, Zimbabwe*

*^2^Department of Chemistry, “Ugo Schiff”, University of Firenze, Via della Lastruccia 3, Sesto Fiorentino 50019, Italy*

*^3^ National Interuniversity Consortium of Materials Science and Technology (INSTM), Via G. Giusti 9, Firenze, FI 50121, Italy*

*^4^ Department of Industrial Chemistry “Toso Montanari”, University of Bologna, Viale del Risorgimento, 4, 40136, Bologna, Italy*

*^5^ Department of Physics and Astrophysics, University of Delhi, New Delhi110007, India*

*^6^ Department of Engineering “Enzo Ferrari”, (DIEF), University of Modena, Via Vivarelli 10, Modena 41125, Italy*

*^7^ Center for Colloid and Surface Science (CSGI), Via della Lastruccia 3, Sesto Fiorentino, FI 50019, Italy*

*^8^Department of Chemical Sciences, Midlands State University, P. Bag 9055, Senga Road, Gweru, Zimbabwe*

Corresponding authors: [wmtangi@cut.ac.zw](mailto:wmtangi@cut.ac.zw), [m.innocenti@unifi.it](mailto:m.innocenti@unifi.it)

^†^‘Author R.K. and Author M.B. contributed equally to this work’

**1) Electrochemical set-up**

**2) CVs MIL53 derivatives**

**3) Ferrocene CV on GC/MIL53 S-CSA (WE)**

**4) CVs Zn-MOF derivatives**

**5) XRD**

**6) SEM images and EDX atom percentage analysis MIL53 Fe MOF derivates**

**7) SEM images and EDX atom percentage analysis Zn-MOF derivates**

**8) DFT: localized orbitals theoretical results**

**9) UV-VIS**

**10)** **Air Mass (AM) experimental spectrum**

**1) Electrochemical set-up**

| 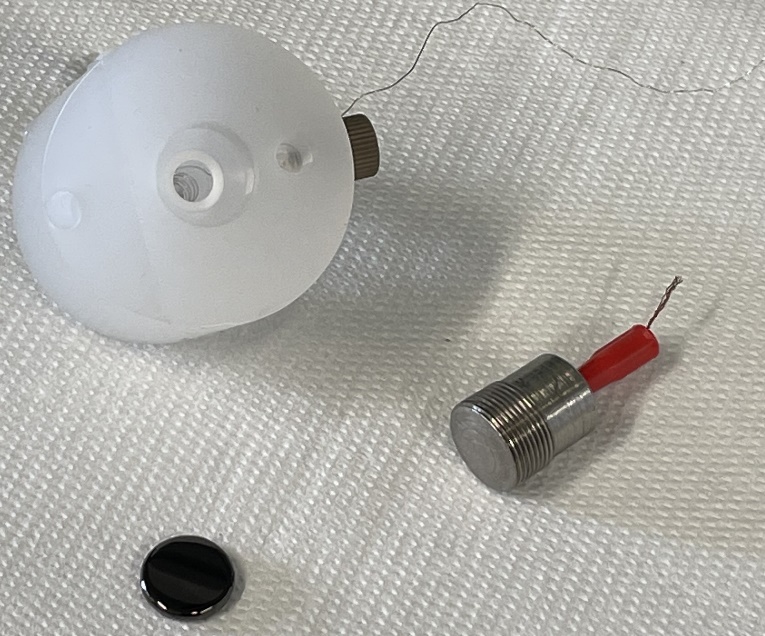 | 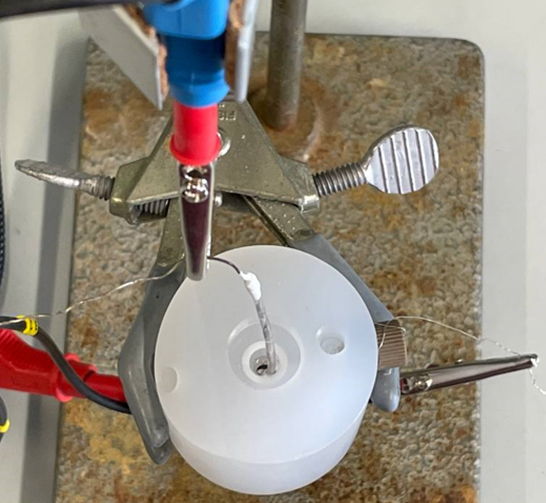 |
| --- | --- |
| a) | b) |

**Figure S1.** Electrochemical cell used in experimental work. a) cell components, b) assembled cell

**2) CVs MIL53 derivatives**

|   a) |   b) |
| --- | --- |
|   c) |   d) |

**Figure S2.** CV curves: in aqueous 0.1 M KCl, Pt (CE), Ag/AgCl/KCl_sat_, 10 mV s^-1^ the potential scan rate, a) GC/MIL53 (WE), b) GC/NH2 MIL53 (WE), c) GC/MIL53 R-CSA (WE), GC/MIL53 L-Cys (WE).

**3) Solid-state CV cross-check**

Figure S3 shows CVs curves for ferrocene and MIL53 S-CSA assembled following the same solid-state set-up procedure as it is used in all the electrochemical CV experiments of this paper. Details about the solid-state cell assembly procedure are given in the main manuscript.

**Figure S3.** Solid-state CVs crosscheck. Red dotted curve ferrocene. Black solid curve MIL53 S-CSA. aqueous 0.1 M KCl, Pt (CE), Ag/AgCl/KCl_sat_, 10 mV s^-1^ the potential scan rate.

**4) CVs Zn-MOF derivatives**

|   a) |   b) |
| --- | --- |
|   c) |   d) |

**Figure S4.** CV curves: in aqueous 0.1 M KCl, Pt (CE), Ag/AgCl/KCl_sat_, 10 mV s^-1^ the potential scan rate, a) GC/Zn-MOF (WE), b) Zn-MOF R-CSA (WE) c) GC/NH2 ZnMOF (WE), d) Zn-MOF L-Cys (WE)

**5) XRD**

| a) |  |
| --- | --- |
| 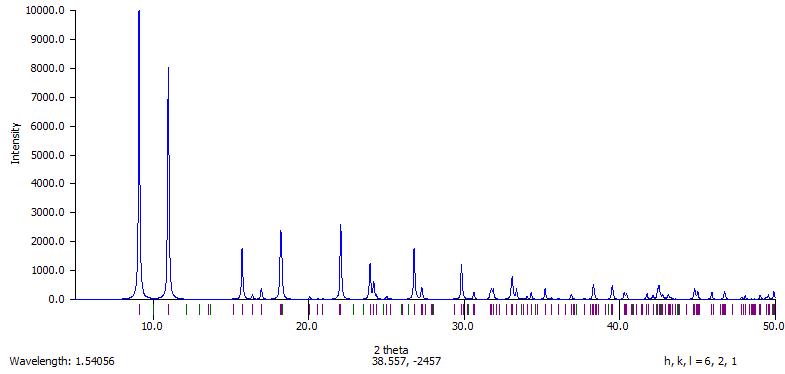 | |
| **a** 19.4223(15) **b** 7.3022(5) **c** 8.8468(7)  **α** 90.00 **β** 90.00 **γ** 90.00  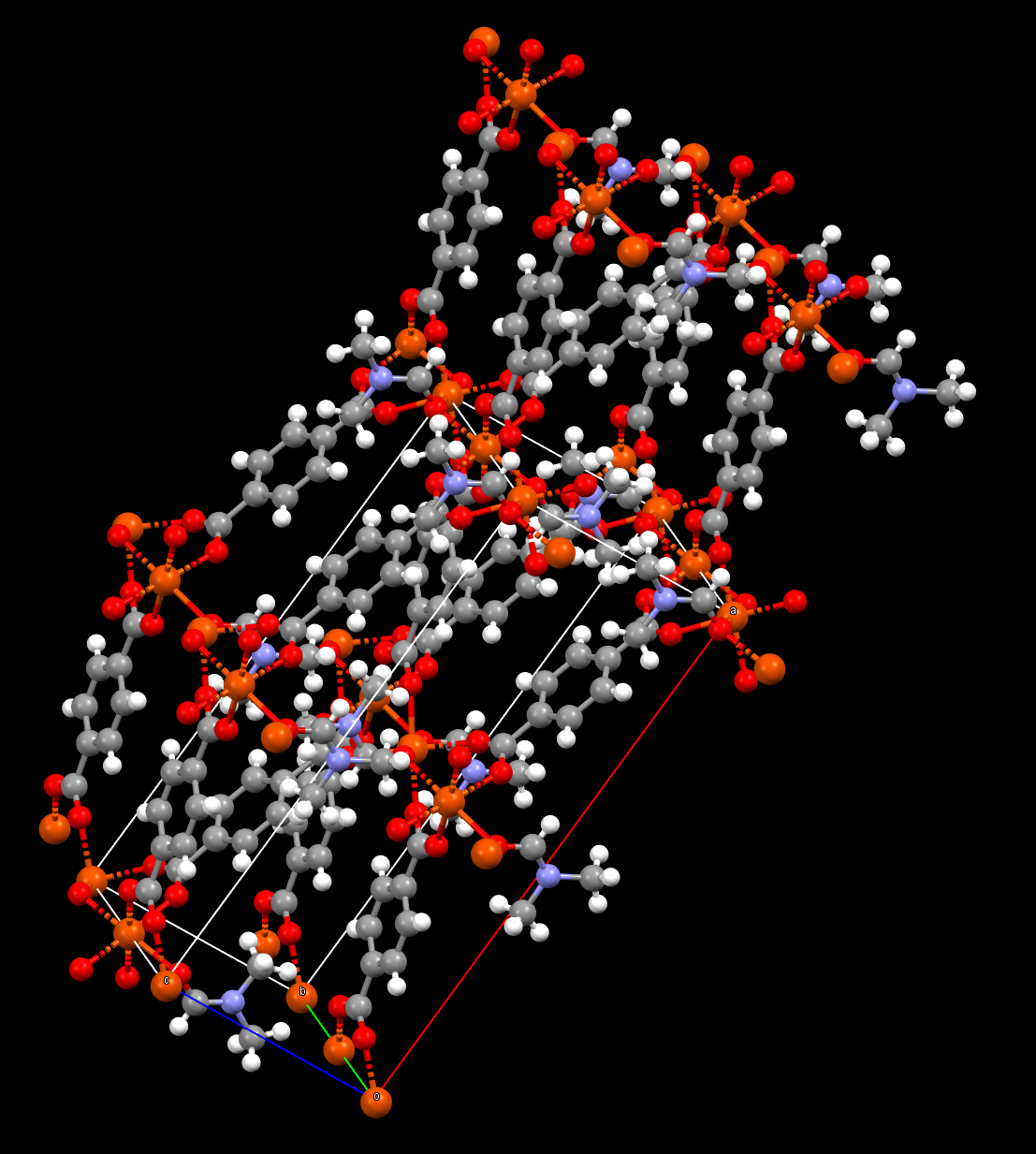  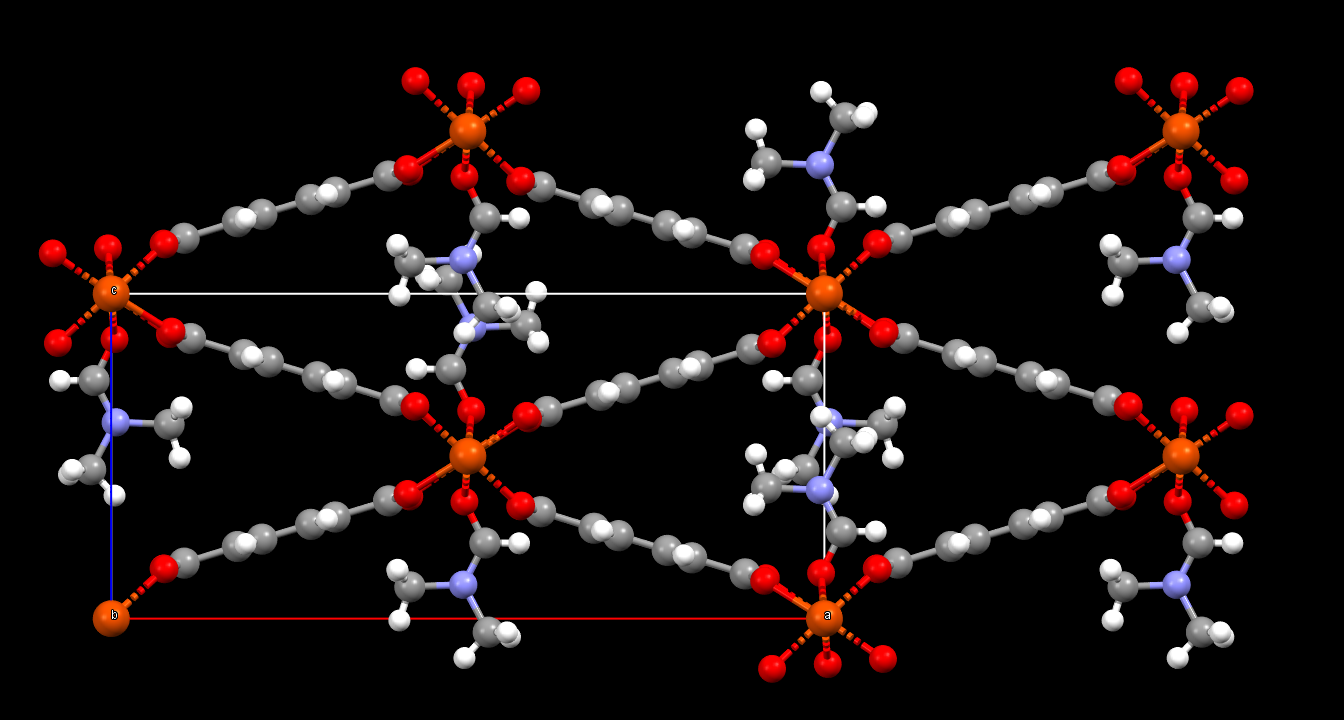 | |
| b) |  |
| 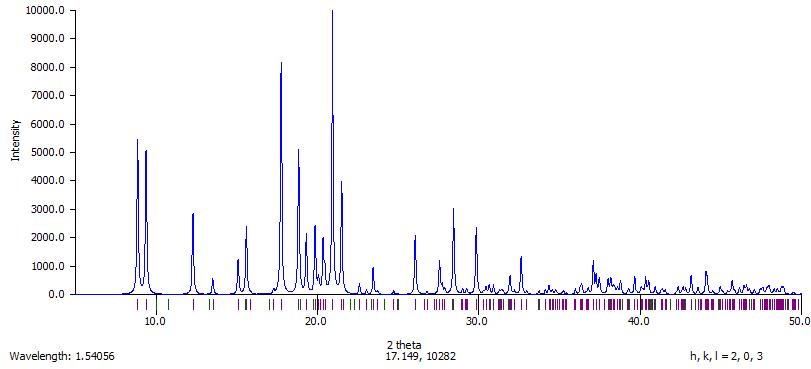 | |
| **a** 18.9102(4) **b** 6.1743(2) **c** 20.0362(4)  **α** 90 **β** 95.453(2) **γ** 90  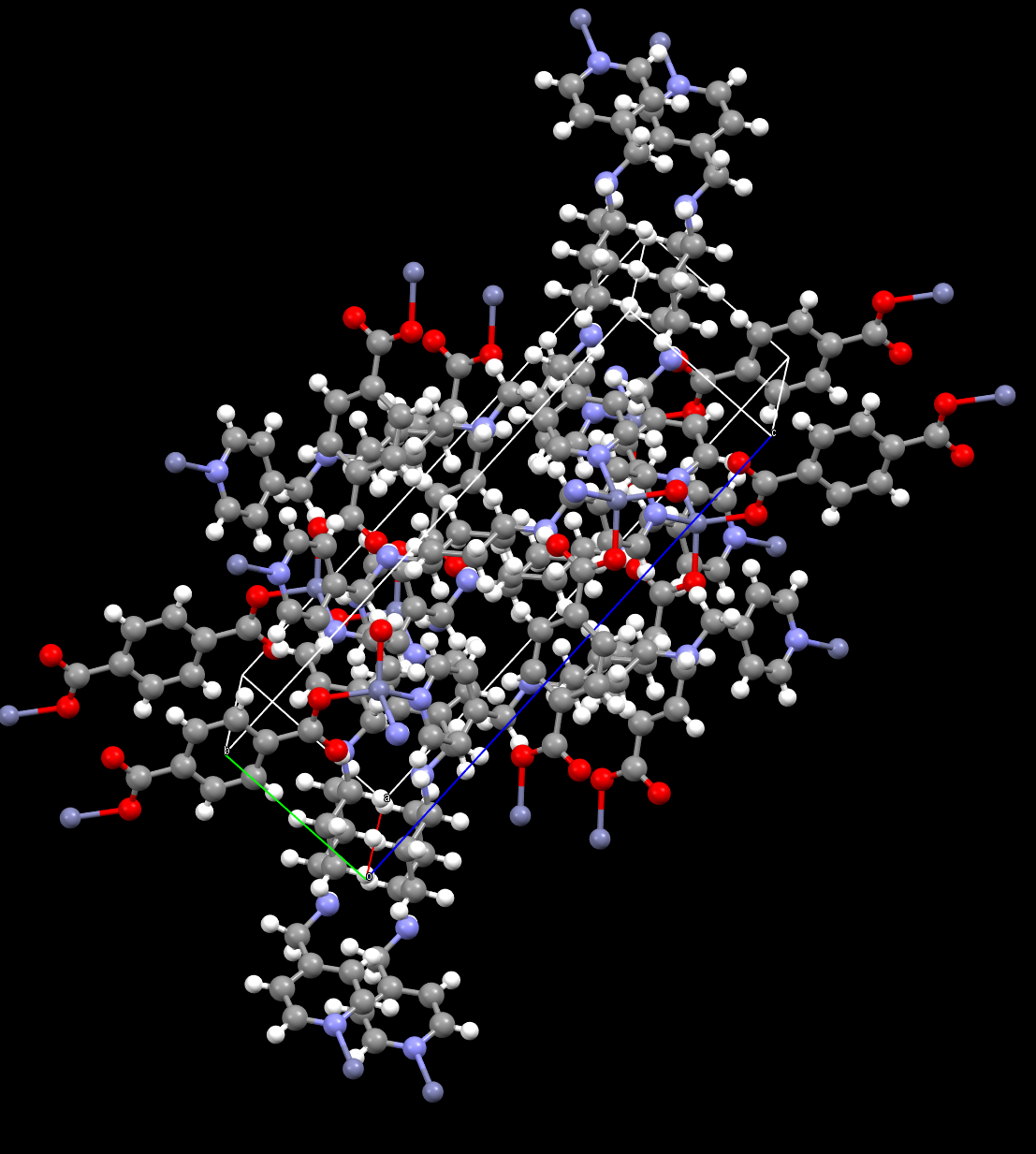  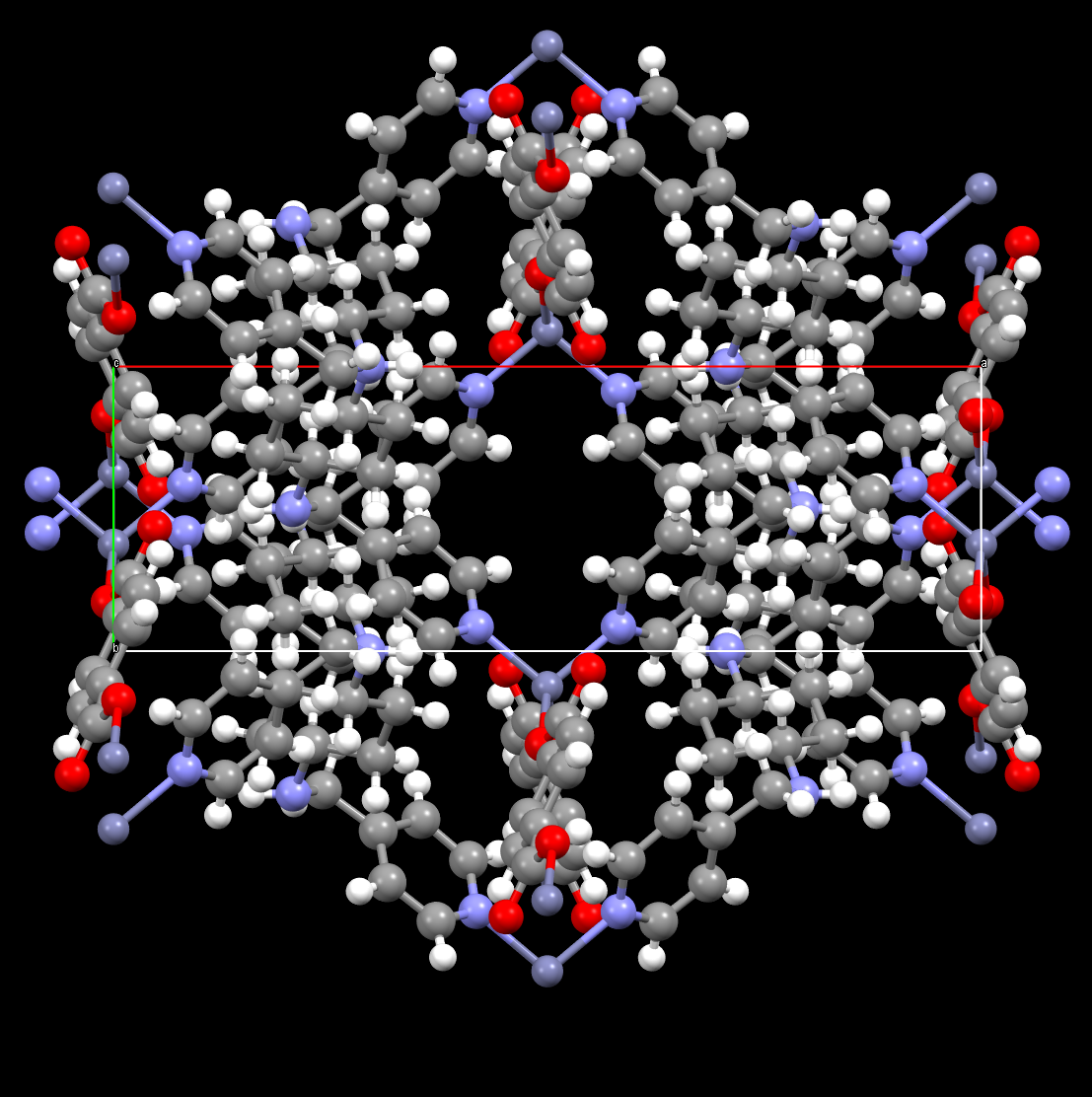 | |

**Figure S5.** calculated theoretical XRD spectra and structure. a) MIL53 b) Zn-MOF

**6) SEM images and EDX atom percentage analysis MIL53 Fe MOF derivates**

Morphological images and chemical elemental analysis (EDS) of the different MIL53 MOF derivatives. See the main manuscript for the details concerning the synthesis and results of the overall physico-chemical characterization.

| 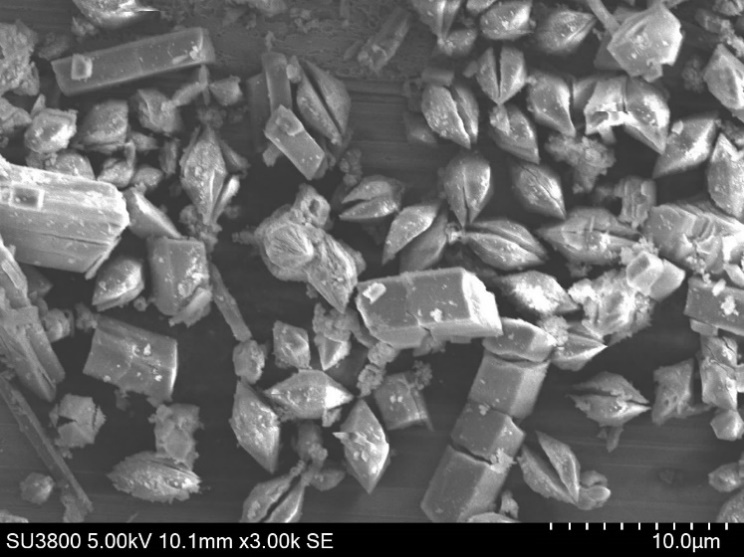  **SE**  **10 µm** | 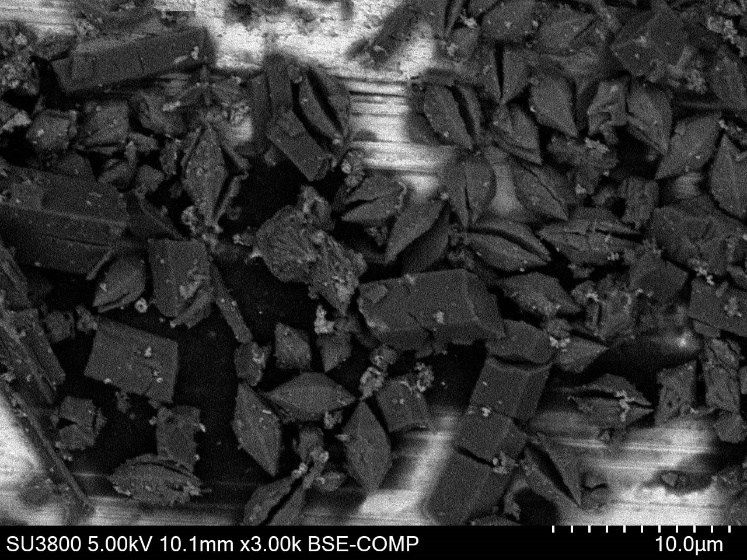  **BSE**  **10 µm** |
| --- | --- |
|  |  |
| 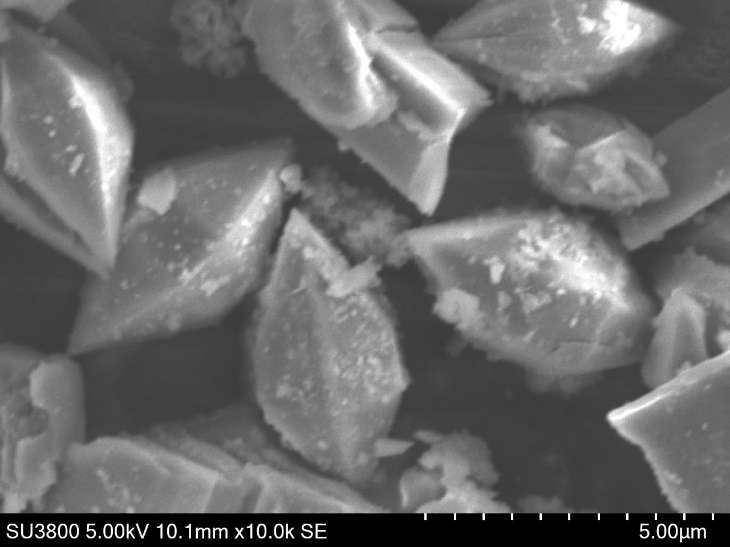  **SE**  **5 µm** | 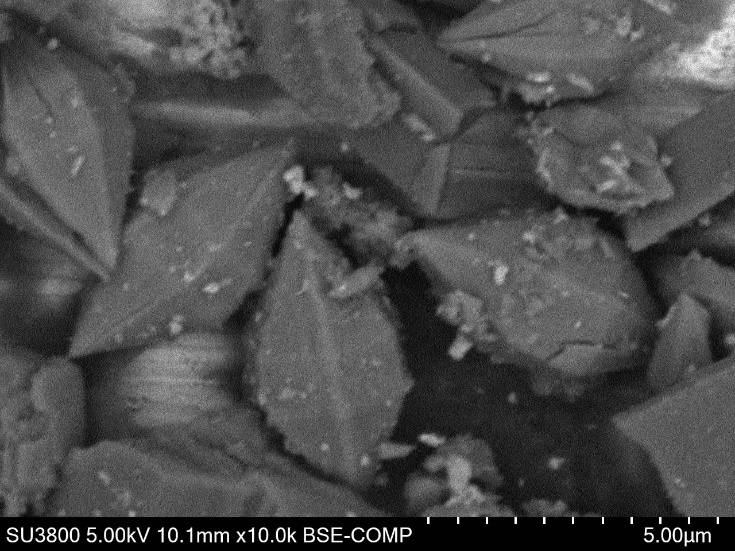  **BSE**  **5 µm** |
| a) | |
|  | |
| b) | |

**Figure S6**. a) SE and BSE SEM images of MIL53, b) EDS spectrum

| 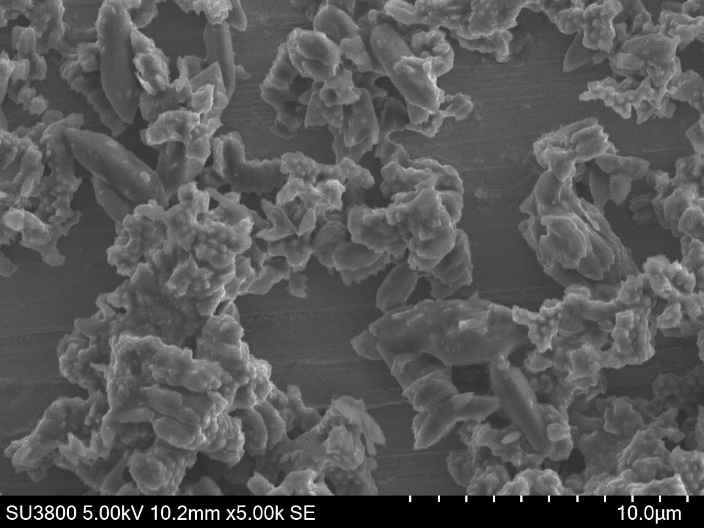  **10 µm**  **SE** | 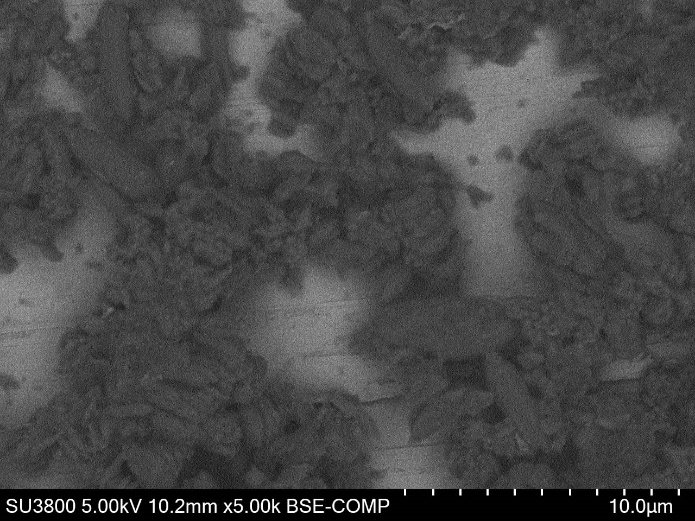  **10 µm**  **BSE** |
| --- | --- |
|  |  |
| 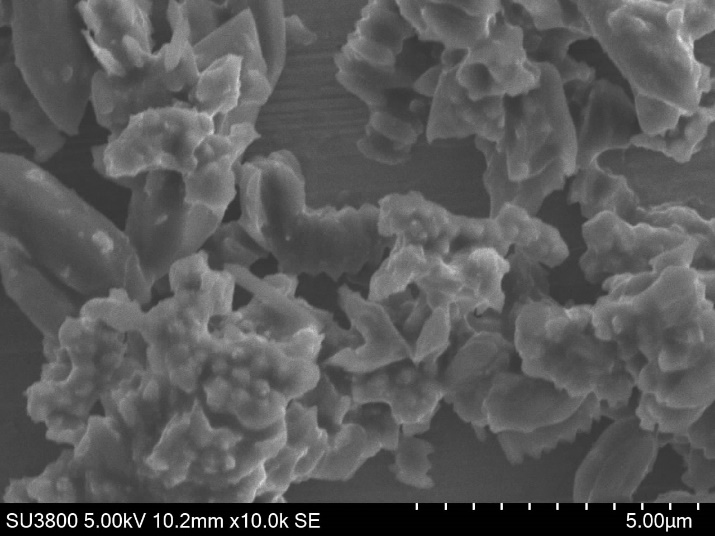  **SE**  **5 µm** | 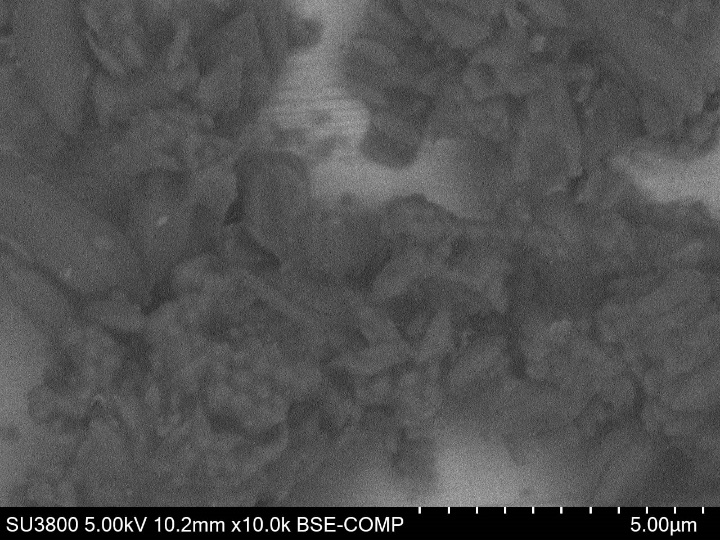  **BSE**  **5 µm** |
| a) | |
|  | |
| b) | |

**Figure S7**. a) SE and BSE SEM images of NH2 MIL53, b) EDS spectrum

| 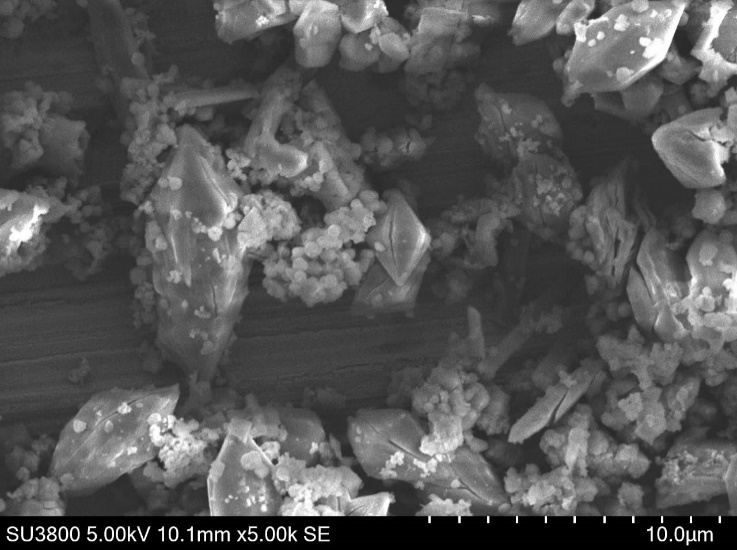  **SE**  **10 µm** | 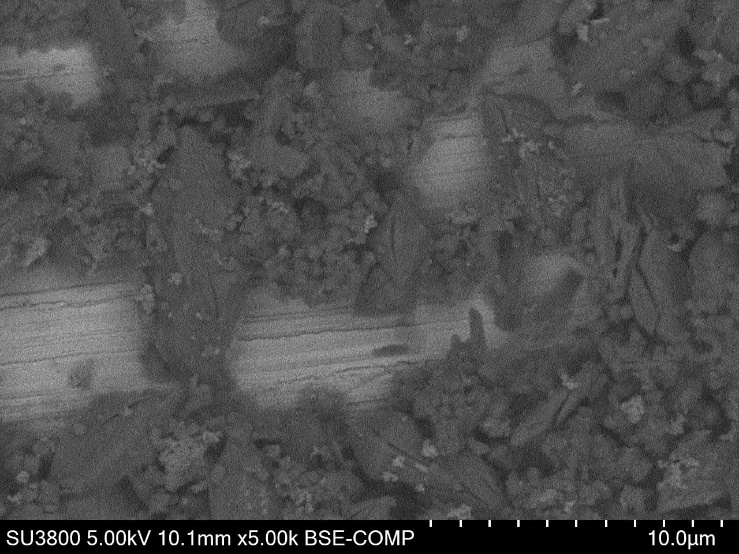  **BSE**  **10 µm** |
| --- | --- |
|  |  |
| 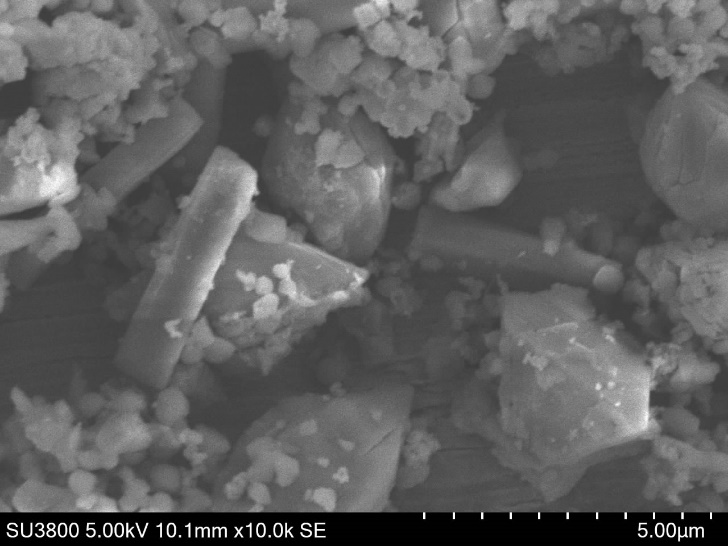  **SE**  **5 µm** | 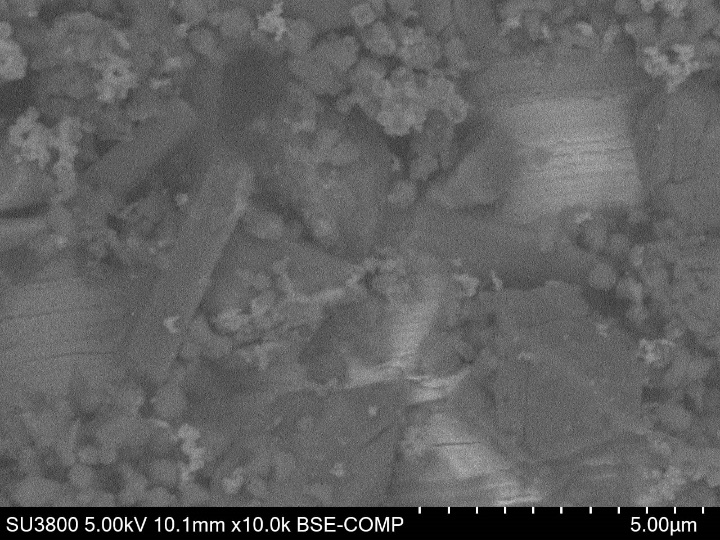  **BSE**  **5 µm** |
| a) | |
|  | |
| b) | |

**Figure S8**. a) SE and BSE SEM images of MIL53 R-CSA, b) EDS spectrum

| 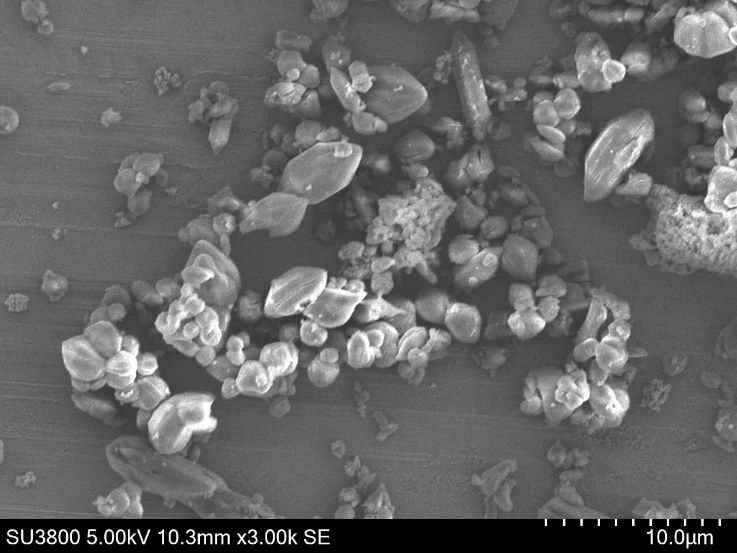  **SE**  **10 µm** | 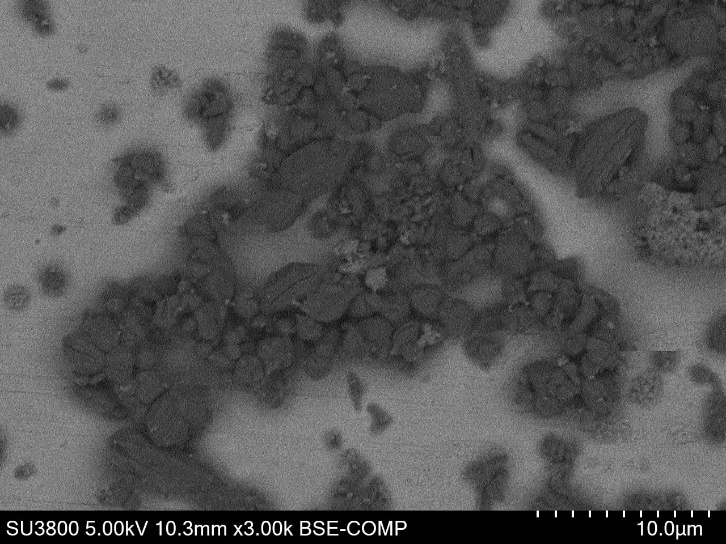  **BSE**  **10 µm** |
| --- | --- |
|  |  |
| 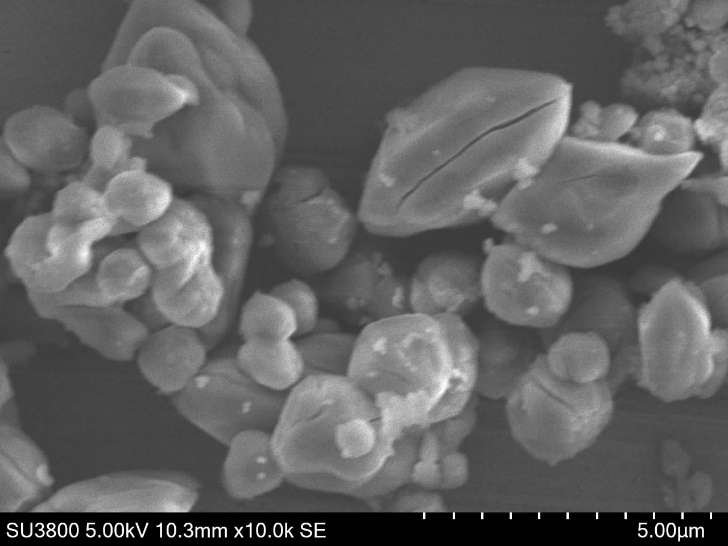  **SE**  **5 µm** | 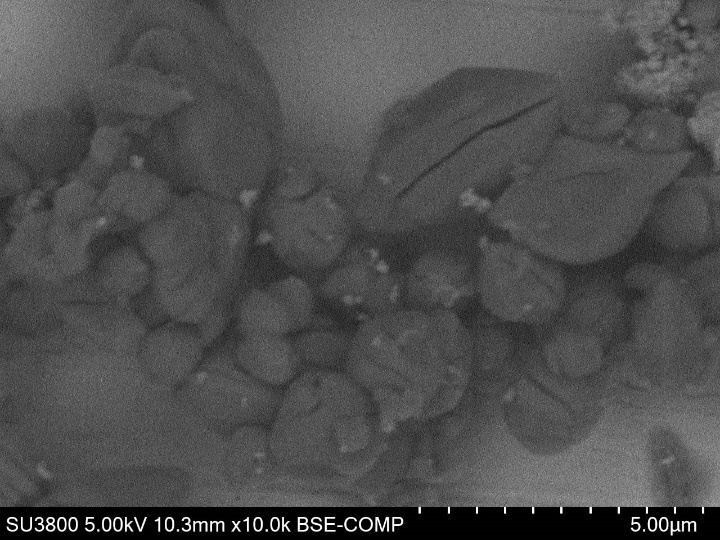  **BSE**  **5 µm** |
| a) | |
|  | |
| b) | |

**Figure S9**. a) SE and BSE SEM images of MIL53 S-CSA, b) EDS spectrum

| 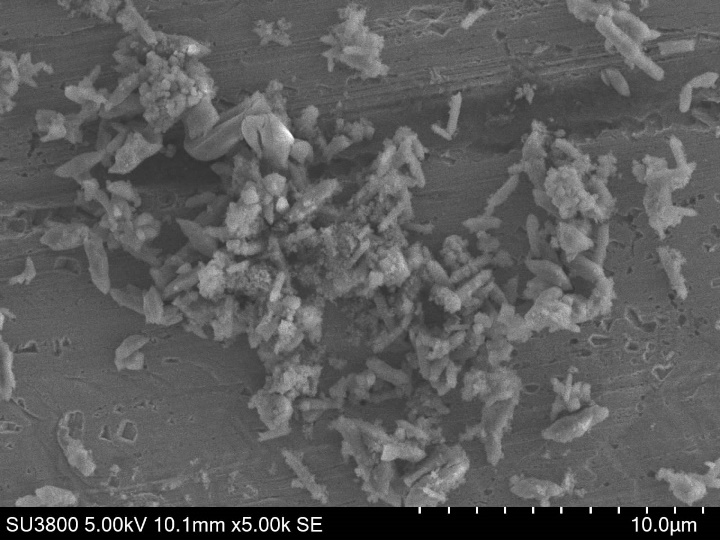  **10 µm** | 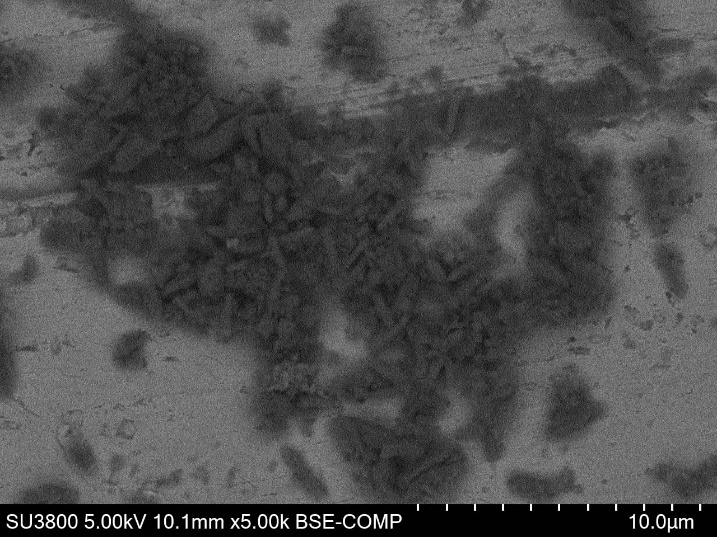  **10 µm**  **BSE** |
| --- | --- |
| **SE** |  |
| 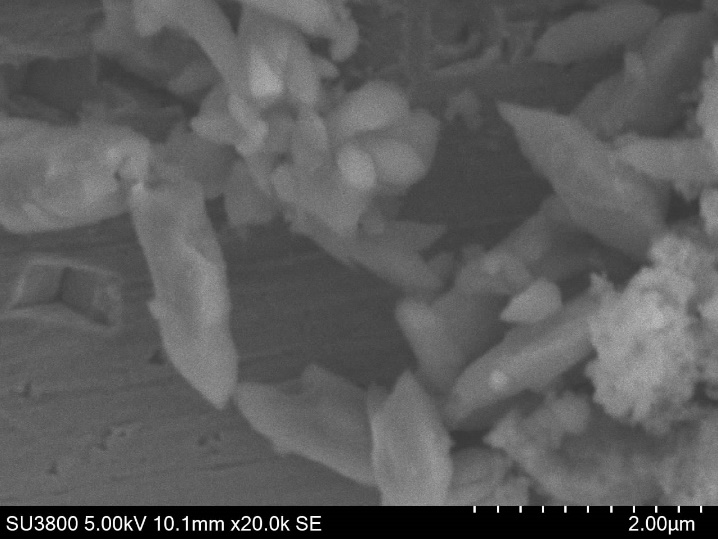  **SE**  **2 µm** | 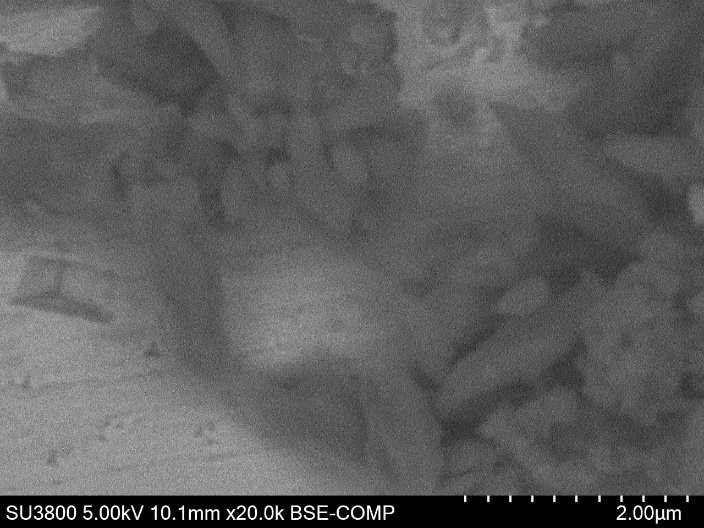  **BSE**  **2 µm** |
| a) | |
|  | |
| b) | |

**Figure S10**. a) SE and BSE SEM images of MIL53 L-Cys, b) EDS spectrum

**7) SEM images and EDX atom percentage analysis Zn-MOF derivates**

| 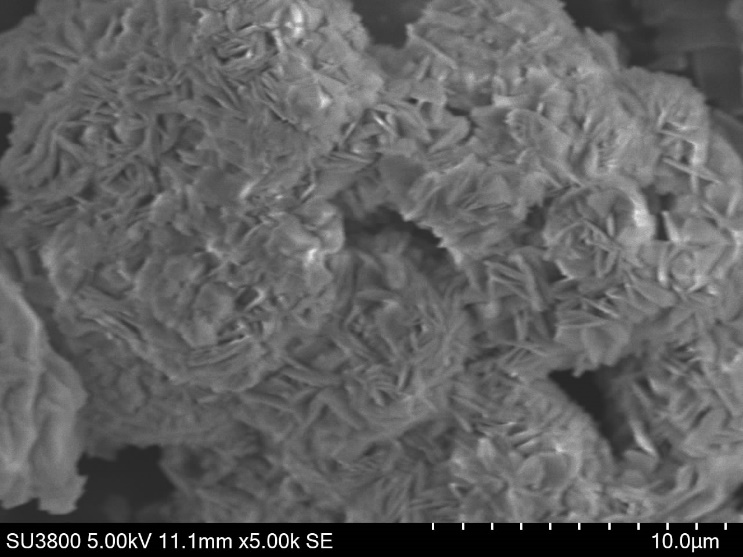  **SE**  **10 µm** | 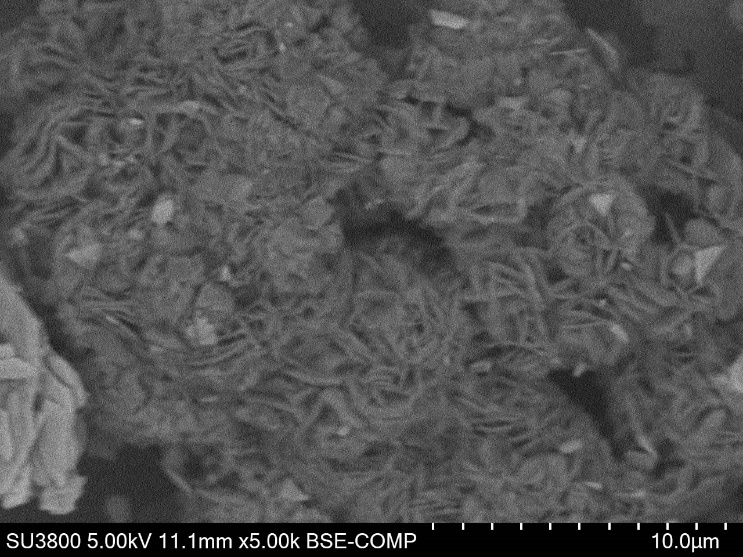  **BSE**  **10 µm** |
| --- | --- |
|  |  |
| 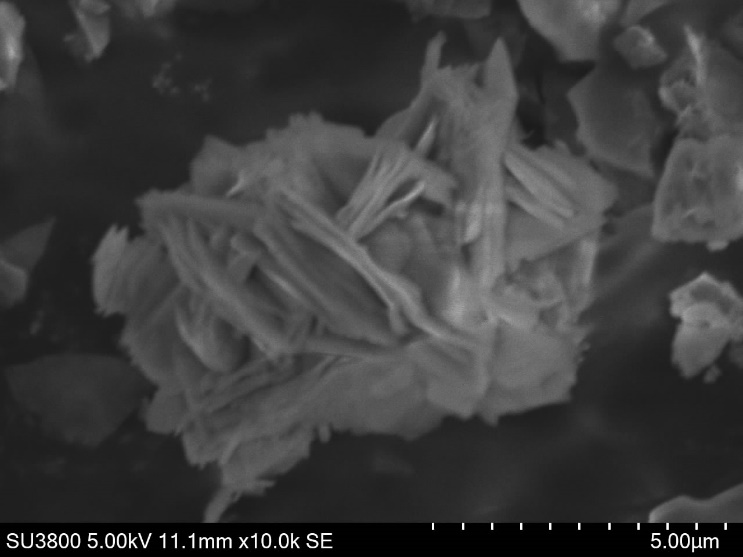  **SE**  **5 µm** | 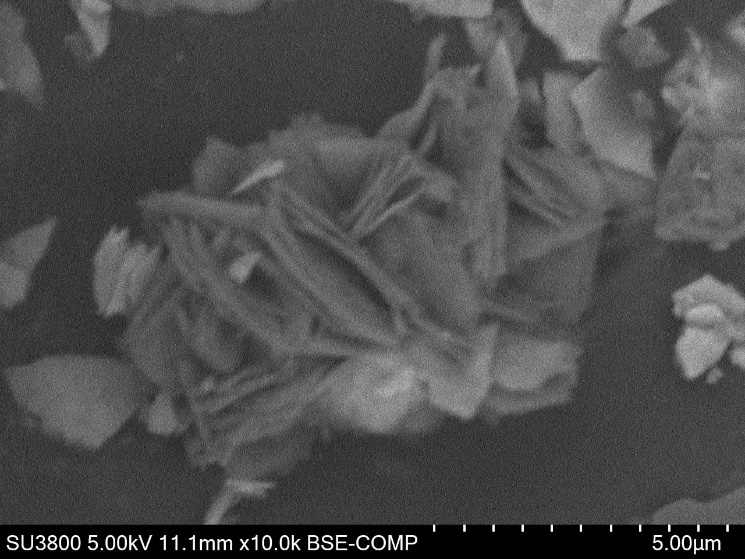  **BSE**  **5 µm** |
| a) | |
|  | |
| b) | |

**Figure S11**. a) SE and BSE SEM images of Zn-MOF, b) EDS spectrum

| 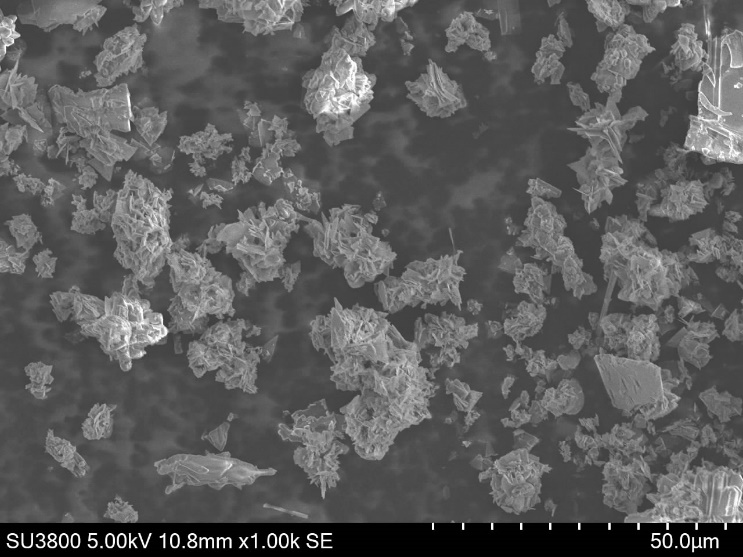  **SE**  **50 µm** | 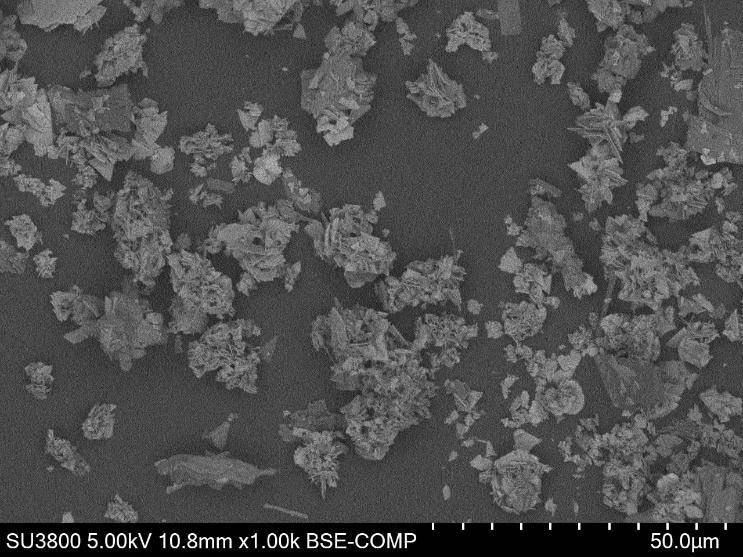  **BSE**  **50 µm** |
| --- | --- |
|  |  |
| 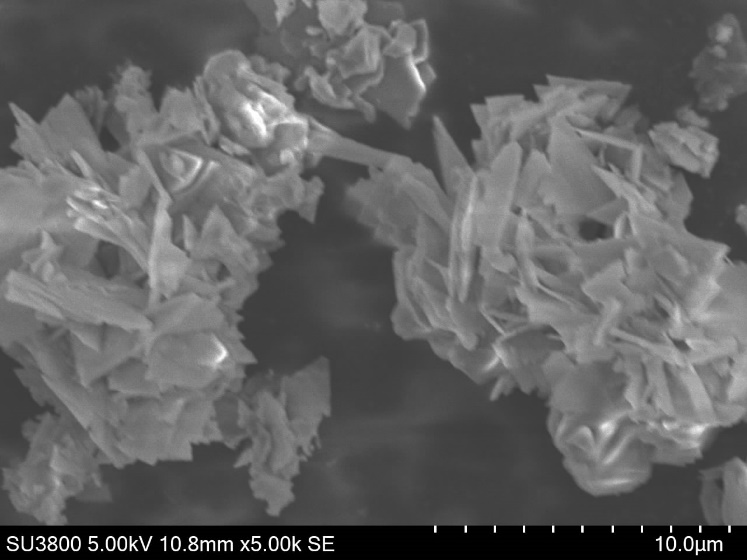  **SE**  **10 µm** | 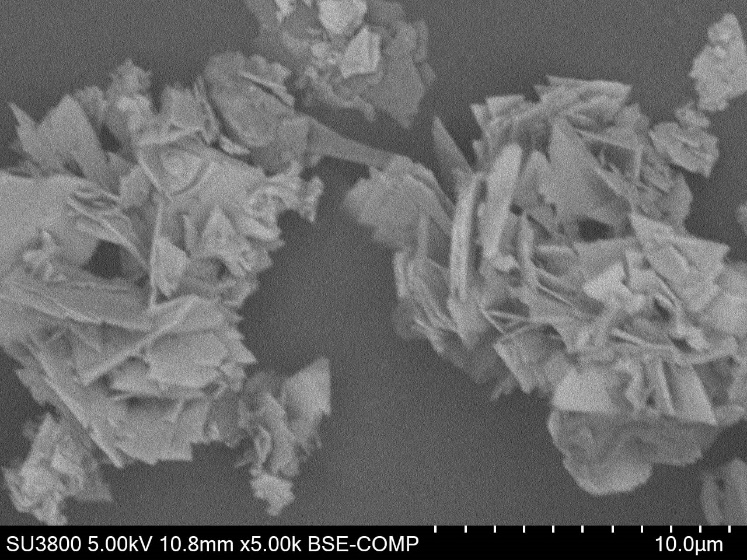  **BSE**  **10 µm** |
| a) | |
|  | |
| b) | |

**Figure S12**. a) SE and BSE SEM images of Zn-MOF S-CSA, b) EDS spectrum

| 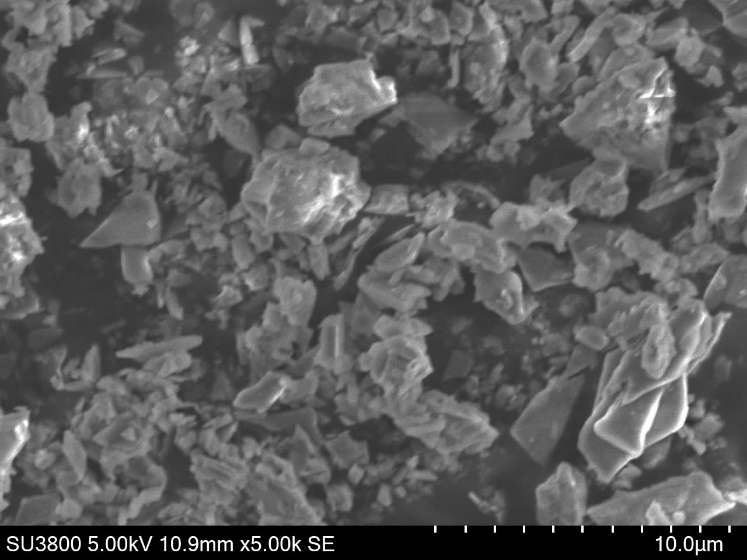  **SE**  **10 µm** | 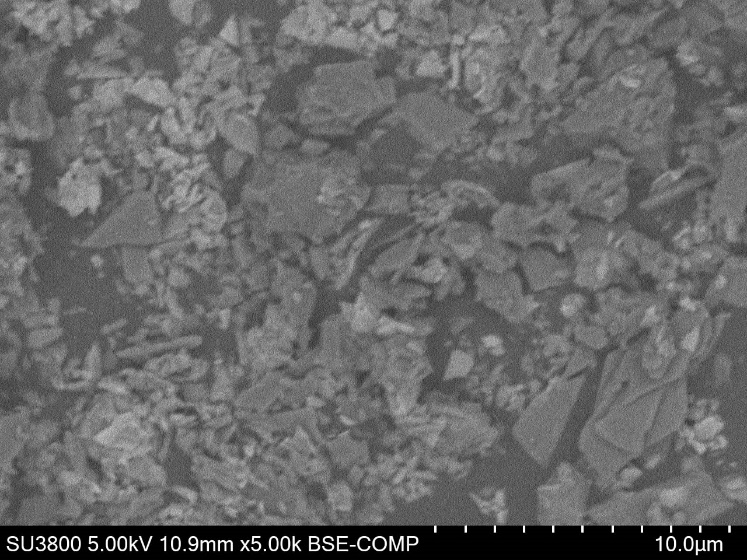  **BSE**  **10 µm** |
| --- | --- |
|  |  |
| 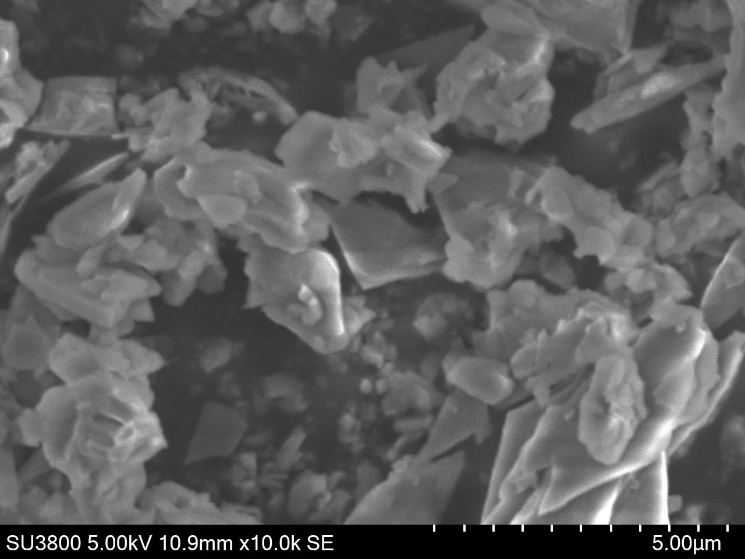  **SE**  **5 µm** | 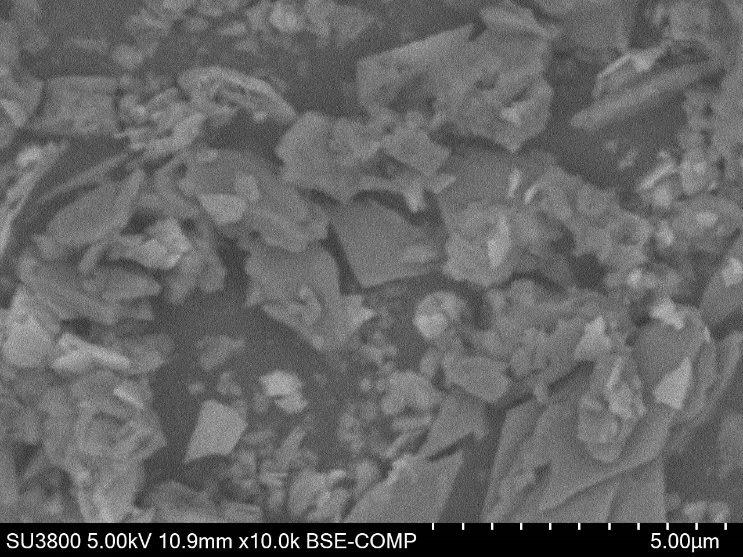  **BSE**  **5 µm** |
| a) | |
|  | |
| b) | |

**Figure S13**. a) SE and BSE SEM images of Zn-MOF R-CSA, b) EDS spectrum

| 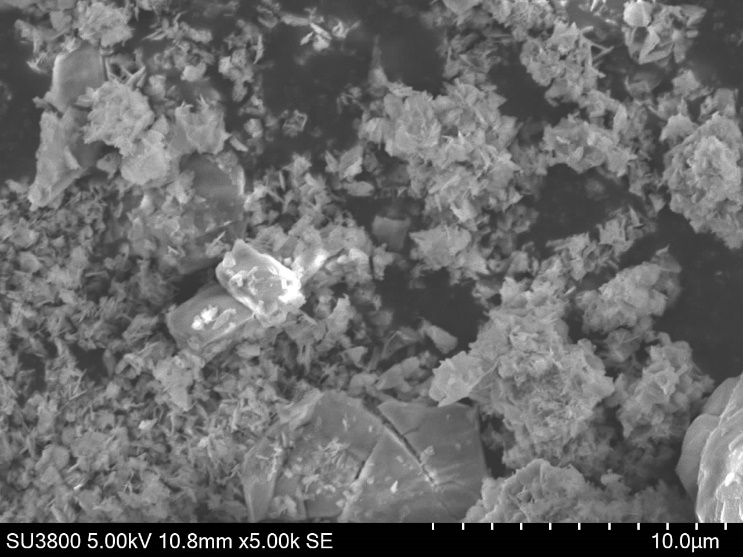  **SE**  **10 µm** | 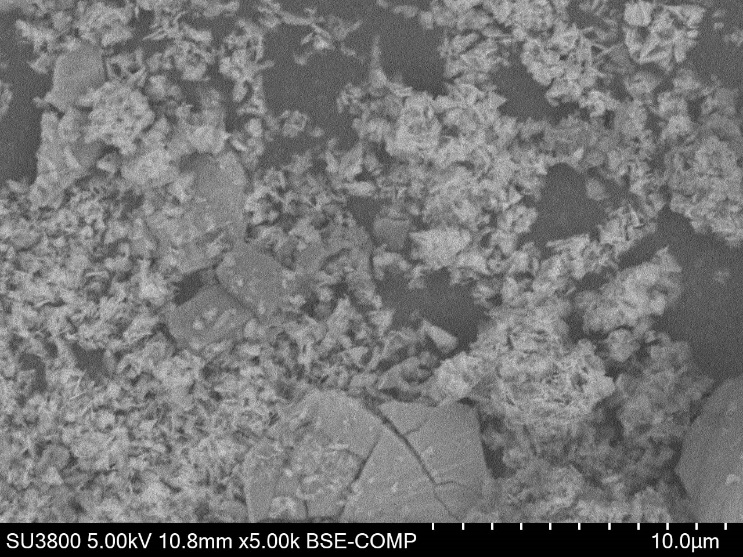  **10 µm** |
| --- | --- |
|  | **BSE** |
| 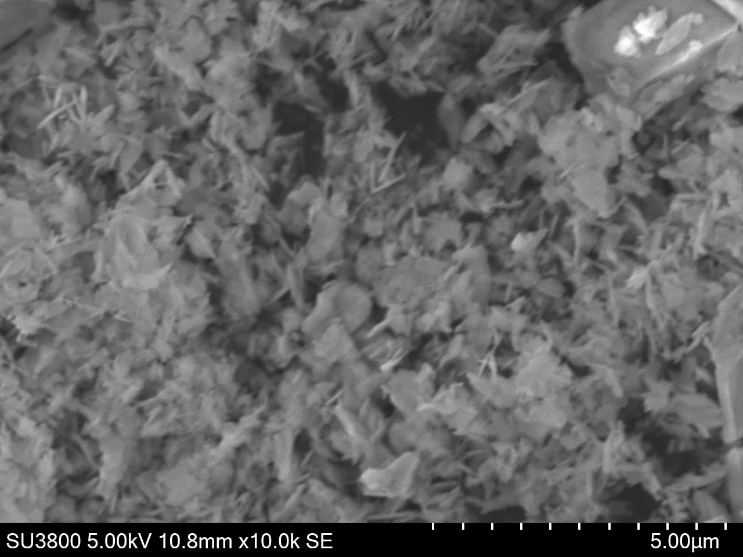  **5 µm** | 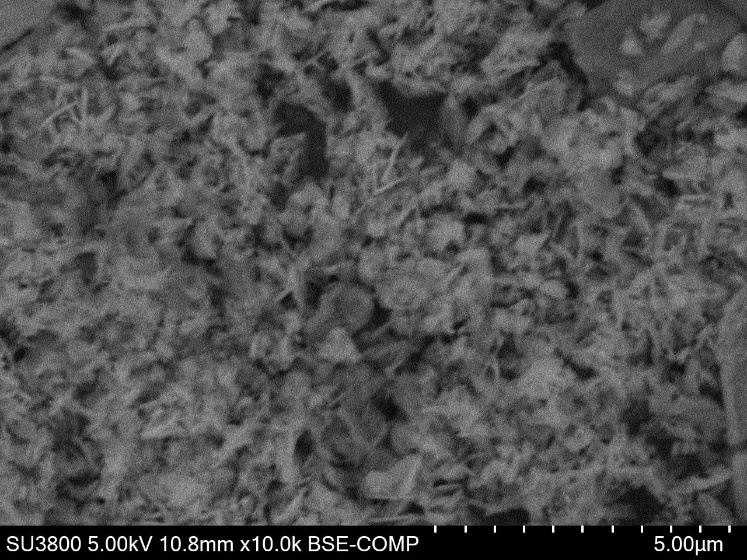  **BSE**  **5 µm** |
| a)  **SE** | |
|  | |
|  | |
| b) | |

**Figure S14**. a) SE and BSE SEM images of NH2 Zn-MOF, b) EDS spectrum

| 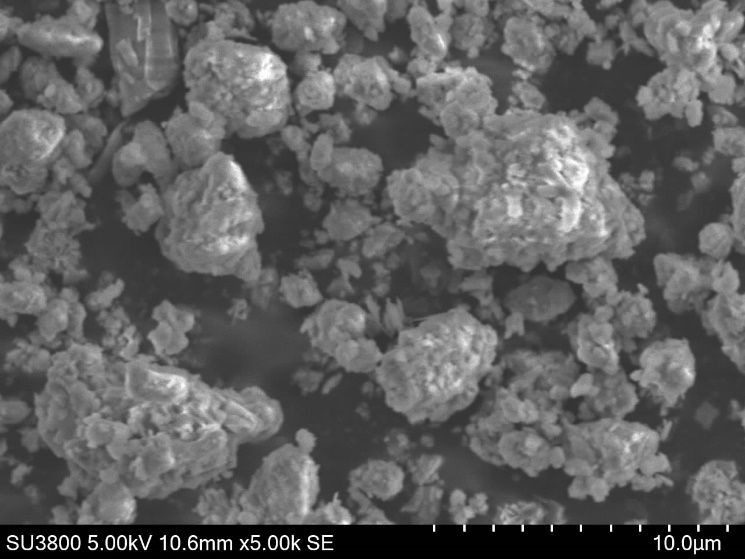  **SE**  **10 µm** | 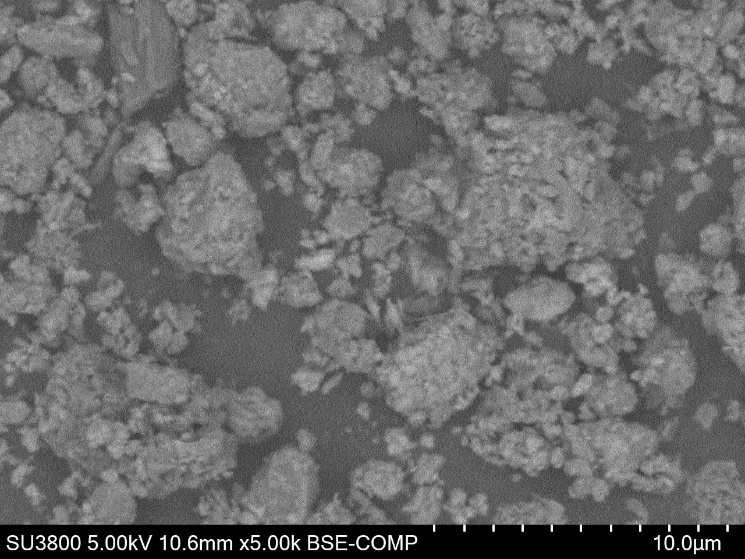  **BSE**  **10 µm** |
| --- | --- |
|  |  |
| 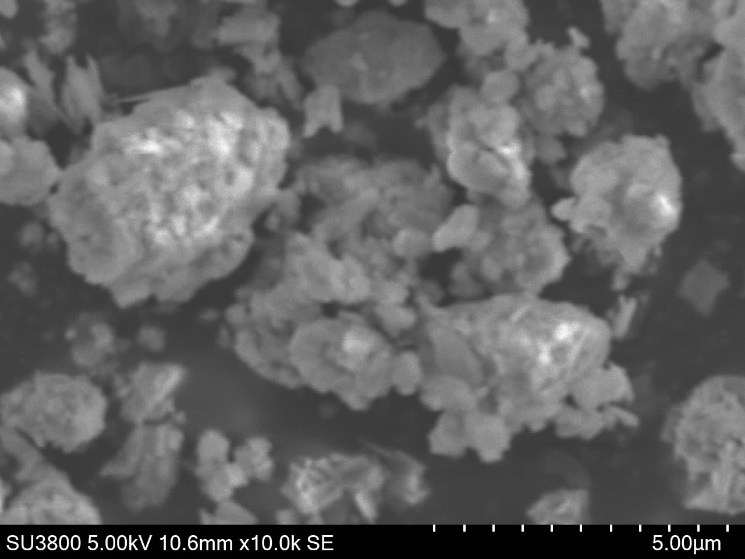  **SE**  **5 µm** | 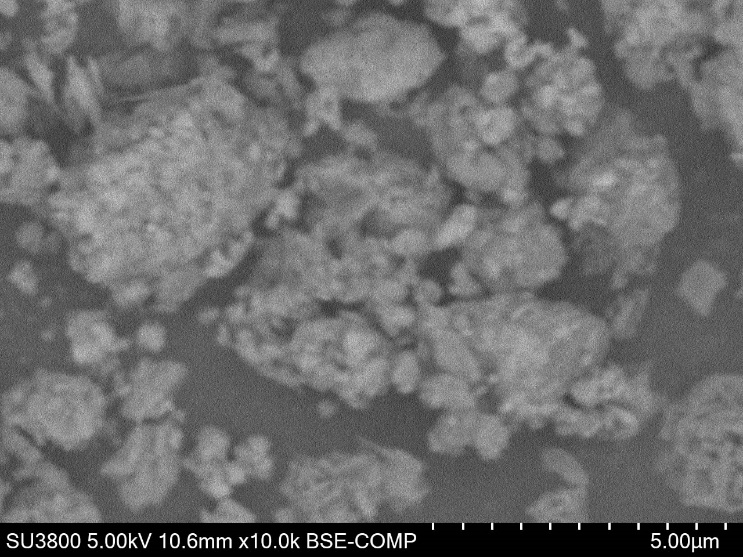  **5 µm** |
| a)  **BSE** | |
| **** | |
| b) | |

**Figure S15**. a) SE and BSE SEM images of Zn-MOF L-Cys, b) EDS spectrum

**8) DFT: localized orbitals theoretical results**

MIL53 modeled as a “supermolecule” constituted by one iron and 4 coordination terephthalic acid molecules. Geometry from the experimental XRD analysis.

|  | Ab-initio DFT  B3LYP/6-31G(d) | Semiempirical  PM6 |
| --- | --- | --- |
| Fe Mulliken atomic net charge | 1.15 | 1.58 |
| HOMO / eV | -5.6851 | -2.8741 |
| LUMO / eV | -3.4819 | -1.1326 |
| HOMO LUMO Gap / eV | +2.2032 | +1.7415 |

Molecular orbital charge density distribution.

Ab-initio DFT: B3LYP/6-31G(d)

| HOMO  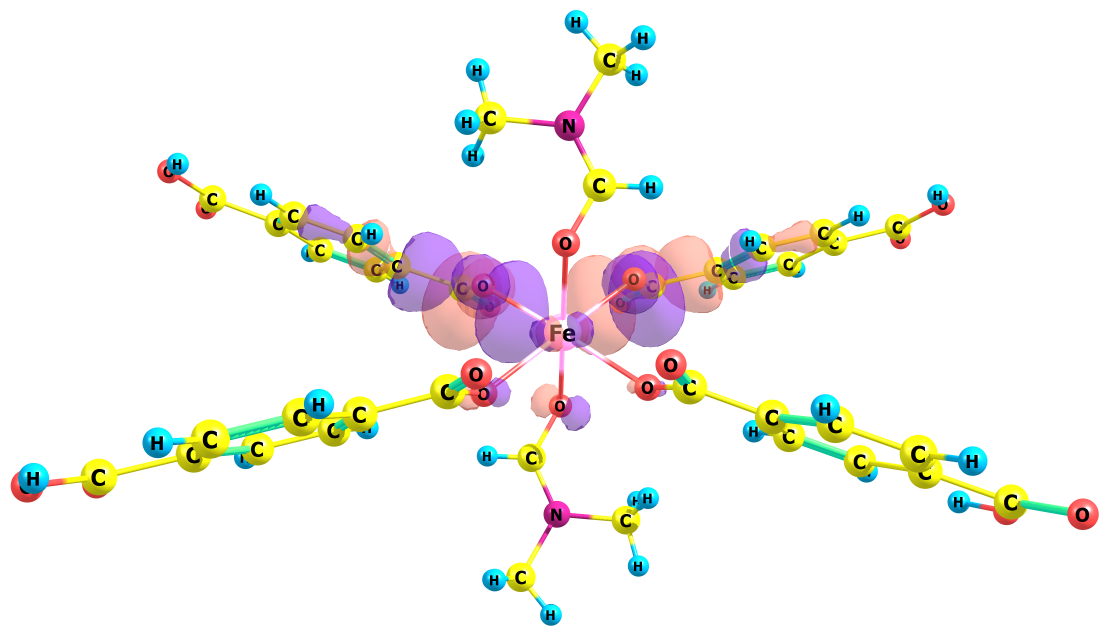 | LUMO  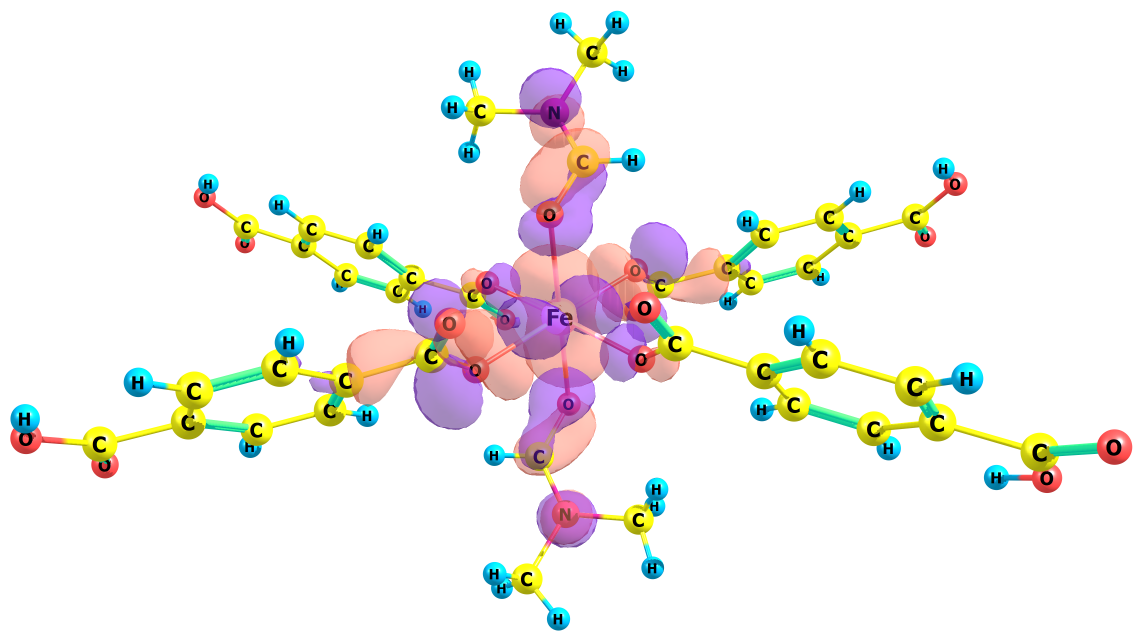 |
| --- | --- |

Semiempirical: PM6

| HOMO  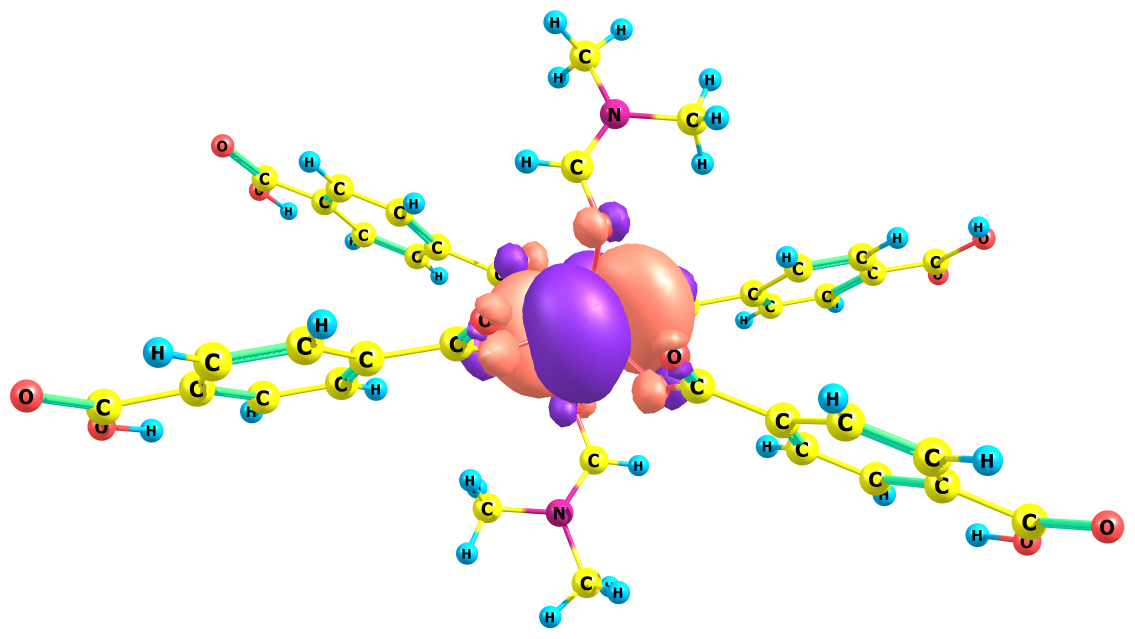 | LUMO  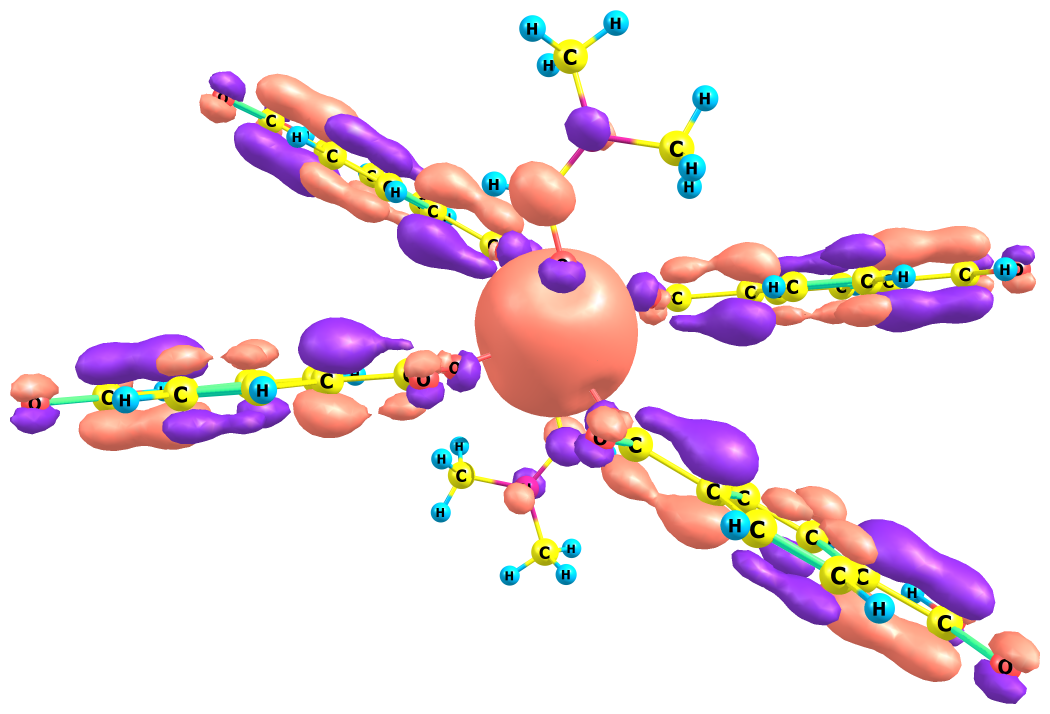 |
| --- | --- |

Remarkably, the HOMO is mainly distributed on the iron cation with a clear and strong delocalization on the surrounding oxygen atoms.

**9) UV-VIS**

**UV-vis spectra of MIL53 and derivatives**


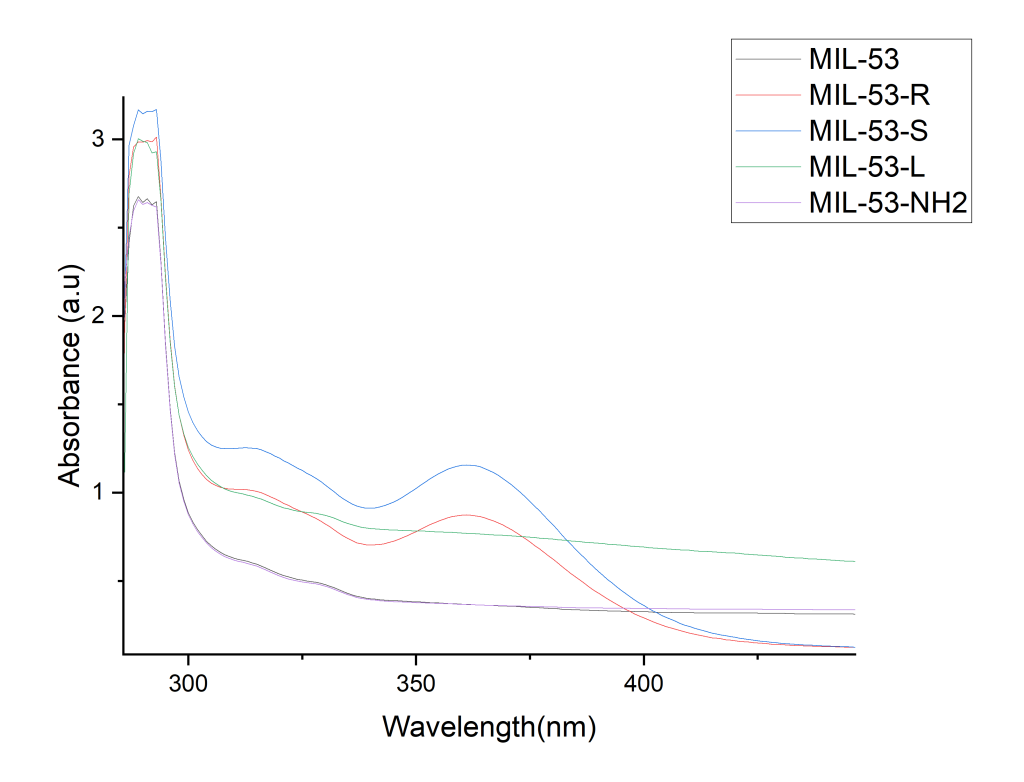


**Figure S16.** Uv-Vis Spectra of MIL53 and its derivatives

**UV-vis spectra of Zn-MOF**


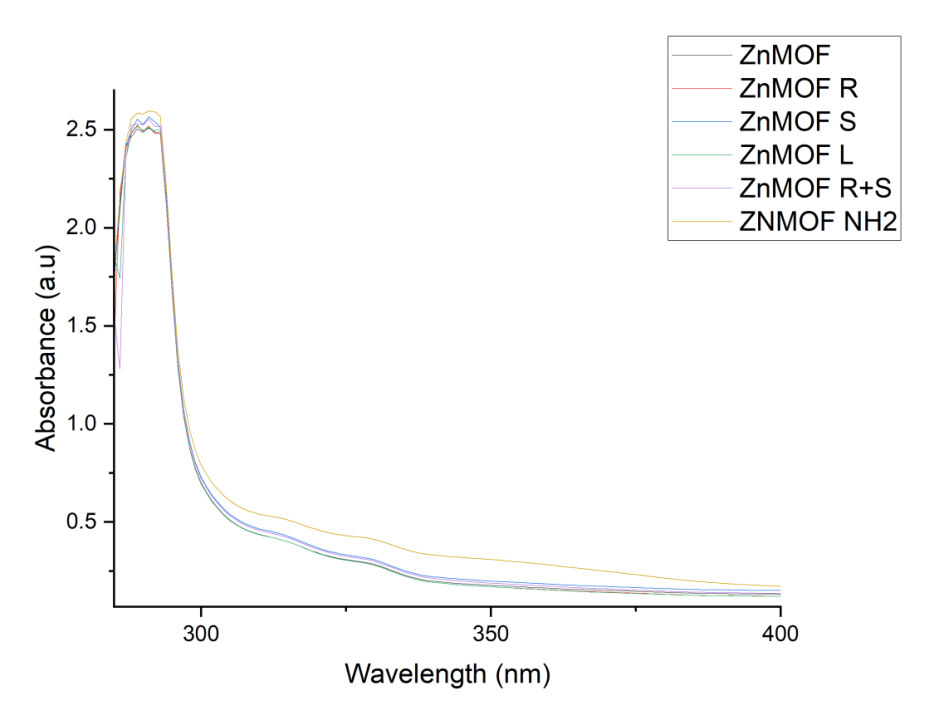


**Figure S17** Uv- Vis Spectra of Zn-MOF and its derivatives

**10)** **Air Mass (AM) experimental spectrum**

Figure S18 shows the Air Mass (AM) experimental spectrum used as excited light source in photoelectrochemical experiments.


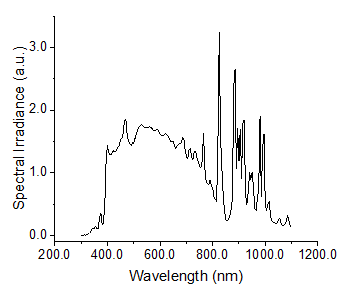


**Figure S18.** AM1.5g experimental spectrum.
